# Adipose tissue is a source of regenerative cells that augment the repair of skeletal muscle after injury

**DOI:** 10.1038/s41467-022-35524-7

**Published:** 2023-01-05

**Authors:** Quentin Sastourné-Arrey, Maxime Mathieu, Xavier Contreras, Sylvie Monferran, Virginie Bourlier, Marta Gil-Ortega, Enda Murphy, Claire Laurens, Audrey Varin, Christophe Guissard, Corinne Barreau, Mireille André, Noémie Juin, Marie Marquès, Benoit Chaput, Cédric Moro, Donal O’Gorman, Louis Casteilla, Amandine Girousse, Coralie Sengenès

**Affiliations:** 1 RESTORE, Research Center, Team 1 STROMAGICS, Université de Toulouse, INSERM, CNRS, EFS, ENVT, Université P. Sabatier, Toulouse, France; 2 Institute of Metabolic and Cardiovascular Diseases, INSERM /Paul Sabatier University UMR 1297, Team MetaDiab, Toulouse, France; 3 School of Health and Human Performance, Dublin City University, Dublin, Ireland; 4 RESTORE, Research Center, Team 2 FLAMES, Université de Toulouse, INSERM, CNRS, EFS, ENVT, Université P. Sabatier, Toulouse, France; 5 RESTORE, Research Center, Team 4 GOT-IT, Université de Toulouse, INSERM, CNRS, EFS, ENVT, Université P. Sabatier, Toulouse, France; 6 Department of Plastic and Reconstructive Surgery, Toulouse University Hospital, 31100 Toulouse, France

**Keywords:** Mesenchymal stem cells, Regeneration, Musculoskeletal development

## Abstract

Fibro-adipogenic progenitors (FAPs) play a crucial role in skeletal muscle regeneration, as they generate a favorable niche that allows satellite cells to perform efficient muscle regeneration. After muscle injury, FAP content increases rapidly within the injured muscle, the origin of which has been attributed to their proliferation within the muscle itself. However, recent single-cell RNAseq approaches have revealed phenotype and functional heterogeneity in FAPs, raising the question of how this differentiation of regenerative subtypes occurs. Here we report that FAP-like cells residing in subcutaneous adipose tissue (ScAT), the adipose stromal cells (ASCs), are rapidly released from ScAT in response to muscle injury. Additionally, we find that released ASCs infiltrate the damaged muscle, via a platelet-dependent mechanism and thus contribute to the FAP heterogeneity. Moreover, we show that either blocking ASCs infiltration or removing ASCs tissue source impair muscle regeneration. Collectively, our data reveal that ScAT is an unsuspected physiological reservoir of regenerative cells that support skeletal muscle regeneration, underlining a beneficial relationship between muscle and fat.

## Abstract (web summary)

The dynamics of fibroadipogenic progenitors (FAPs) after muscle injury are crucial to ensure efficient regeneration. Here the authors show that a pool of FAPs originates from adipose tissue and are necessary for effective muscle regeneration.

## Introduction

Skeletal muscle exhibits a remarkable regenerative capacity in adult mammals and a large effort is underway to better characterize and understand the underlying mechanisms controlling this process. The regenerative potential of skeletal muscle relies on a pool of resident adult stem cells, the satellite cells which proliferate and differentiate to allow muscle growth and remodeling in response to exercise or following trauma^1–3^. In the past decade, studies have identified mesenchymal progenitors, termed “fibroadipogenic progenitors” (FAPs), providing key functional support to satellite cells^4–7^. Besides their supportive role, the regulation of FAP content is also crucial since their absence leads to regeneration impairment, whereas FAP maintenance in the late phase of regeneration leads to fibrosis and/or fatty degeneration of the injured muscle^8–10^.

However, the early evolution of FAP number upon injury is less well characterized. Intriguingly, this process exhibits distinct waves, a first one occurring 1-day post-injury (dpi), when FAP proliferation has not started yet, followed by a second one at 3 dpi, due to FAP proliferation^4,9,11–14^. Without being supported by FAP proliferation, the first wave of FAP content increase is not well understood and raises the question that different FAP subpopulations coming from different sources. In support of this hypothesis, single-cell analysis technology has revealed the heterogeneity of FAPs cell populations^11,15–17^ and also indirectly described the first wave of FAP. Indeed, Oprescu et al. elegantly reported the presence of a subpopulation of “activated” FAPs in the very early phases following muscle injury (0.5 dpi)^17^. These activated FAPs are transcriptionally distinct from the non-injured FAP populations and were considered by the authors to be muscle resident, although direct demonstration was not demonstrated.

White adipose tissue (AT) houses a mesenchymal cell population, which resembles FAPs, the adipose stromal cells (ASCs). Various studies, including ours, show that ASCs exhibit similar cell surface antigen combination, same clonogenic activity, and differentiation potentials^18–20^. ASCs attract a lot of interest due to their high regenerative potential and represent very promising tools for cell-based therapies^21,22^. Whether FAPs and ASCs are distinct cell types remains unclear^23–25^. We previously demonstrated that subcutaneous AT (ScAT) releases ASCs in response to inflammatory stimuli^18,26^ or to high-fat diet^27^. Moreover, we and others showed that intramuscular adipocytes partly arise from these circulating ASCs, particularly upon weight gain^27–29^. Consequently, we questioned here whether the first wave of FAP number increase in response to acute muscle injury is the result of ASCs release from ScAT followed by their further infiltration into the damaged muscle to support its regeneration.

In the present study, we report that ScAT releases ASCs in response to acute muscle damage and that released ASCs infiltrate the injured muscle via a platelet-dependent mechanism. We also show that blunting the infiltration of ASCs impairs muscle regeneration suggesting that ScAT is an unexpected partner of muscle regeneration.

## Results

### FAPs transcriptomic profile resembles ASCs after muscle injury

**Fig. 1:**
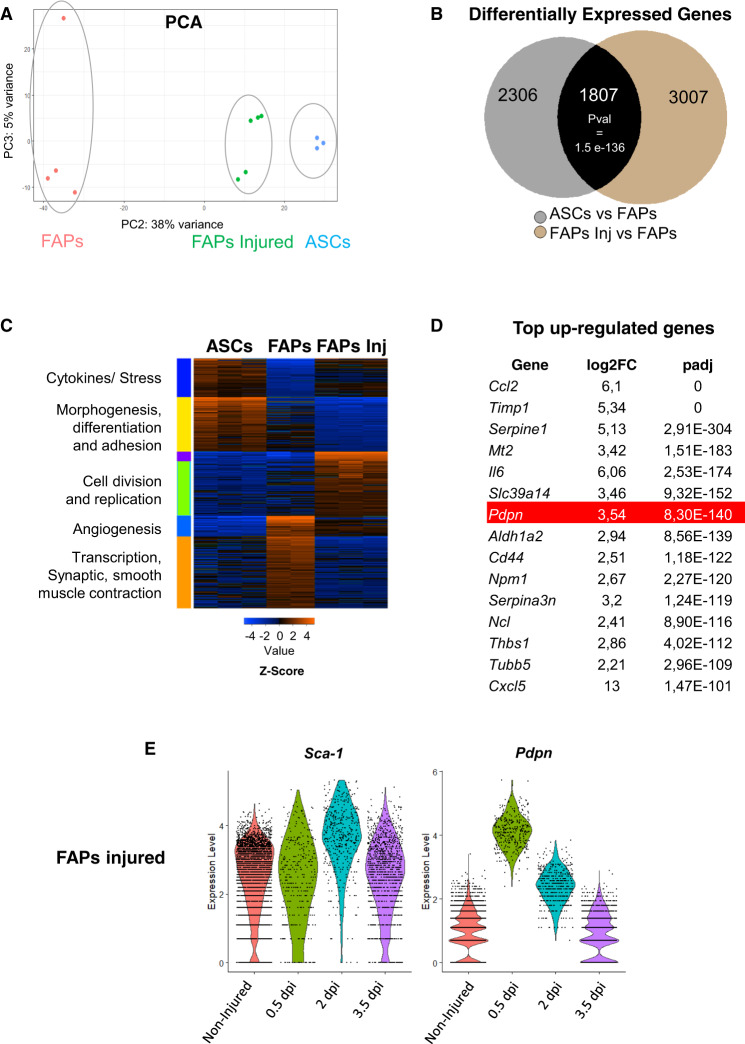
Muscle injured derived FAPs resemble ASCs from ScAT. **A** Principal-component analysis (PCA, PC2 vs PC3) of RNAseq expression values of FAPs and ASCs isolated from injured (1 dpi) and control animals. **B** Venn diagram showing overlap of differentially expressed genes in ASCs from ScAT (SCAT) and FAPs from injured muscle (FAPInj) as compared with FAPs in non-injured animals (FAP). LOG2FC > 0.58 and *p* value<0.05. **C** Expression heatmap of K-mean clustering of differentially expressed genes (expressed in *Z* score) in FAPs groups (control and injured) compared with ASCs and corresponding Gene Ontology terms. **D** Table list of the top upregulated genes in ASCs compared with FAPs ranked by adjusted *p* value. Significance was obtained using DESeq2 package set to default parameters which uses the Wald test to calculate *p* values. **E** Violin plots of *Sca-1* and *Podoplanin* (*pdpn*) of single-cell RNAseq expression of FAPs isolated from injured (0.5, 2, and 3.5 dpi) and control animals using datasets from Oprescu et al.^17^.

We first compared the transcriptomic profiles of ScAT-derived ASCs to the one of non-injured or injured FAPs (1 day post-injury, dpi) using RNAseq analysis. To strengthen our data, we included data from Malecova et al.^15^ for FAPs and injured FAPs on day 1. Principal-component analysis (PCA) showed that replicates for each condition were gathered in distinct groups (Figure S1A). Injured FAPs (1 dpi) were further separated from ASCs along PC1 (54% of variance). Interestingly, however, they were much closer to ASCs rather than with non-injured FAPs along the PC2 component that accounts for 38% variance (Fig. 1A). Moreover, analysis of differentially expressed genes, compared to uninjured FAPs, showed convergence towards largely overlapping transcriptional landscapes between injured FAPs and ASCs at 1 dpi (*P*val = 1.56 e-136, Figs. 1B, 1C). This similarity in the composition of transcriptomes could be due to the convergence of the function of activated FAPs and ASCs. It also raises the possibility that ASCs from adjacent ScAT could infiltrate muscle. To potentially identify a selective marker of this FAP population, we analyzed top-upregulated genes at 1 dpi and found that Podoplanin, recently shown to impart a pro-migratory phenotype in bone marrow-derived mesenchymal stromal cells (MSCs)^30^, was the seventh-most up-regulated gene (Fig. 1D). Then we used a published scRNA seq dataset to monitor the dynamics of Podoplanin expression in the FAP cell populations during muscle regeneration, particularly in the early stages following injury. We used the dataset from the Oprescu et al.^17^ study, which explored the transcriptional dynamics of various cellular subpopulations during the skeletal muscle regeneration process and particularly in the very early phases following injury. FAPs, defined as Sca-1 (Ly6a)-positive cells, were marked by the expression of Podoplanin at an early regeneration stage (0.5 dpi) before a transition back to basal levels at 3.5 dpi (Fig. 1E). Importantly, it has been recently reported in bone marrow MSCs that Podoplanin expression confers a pro-migratory phenotype facilitating their intravasation across the vessel wall and interaction with circulating platelets^30^. Consequently, these data show that a subpopulation of FAPs following muscle injury resembles ASCs, thus suggesting that they may originate from AT.

### Muscle injury causes a FAP rise mirrored by a ScAT ASC decrease

**Fig. 2:**
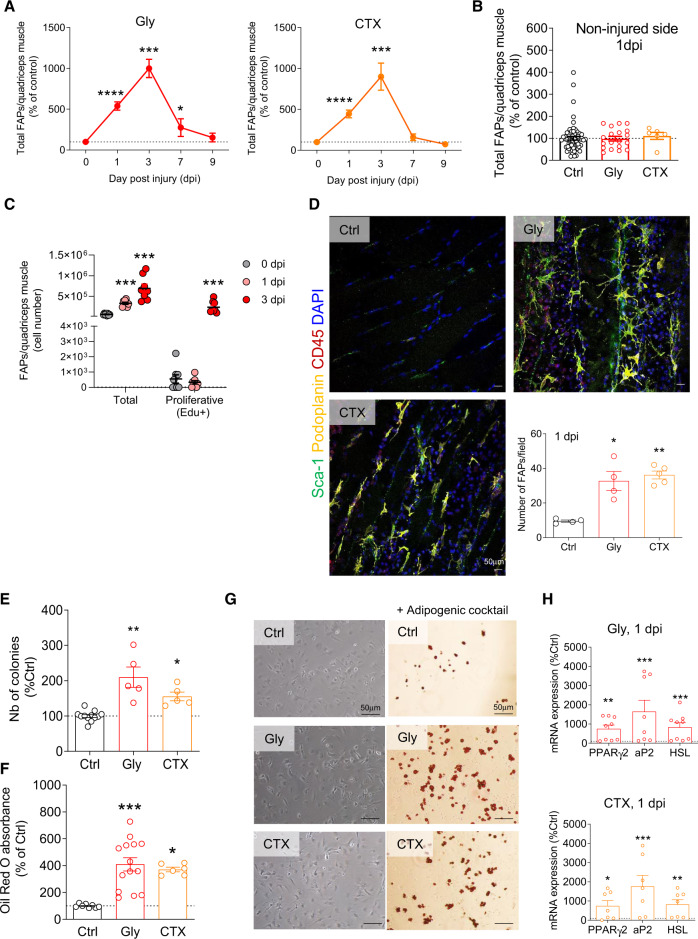
FAPs content increases within 24 h after muscle injury. **A**, **B** Myofiber damage was induced by intramuscular injection of glycerol (Gly) or cardiotoxin (CTX) into the quadriceps. FAP number was quantified in quadriceps-derived SVF by flow cytometry (with the markers CD31, CD45, Sca-1, CD34, CD140α, and podoplanin) from 1 to 9 dpi and compared between control (uninjured, Ctrl), Gly or CTX injected animals (**A**, **B**) as well as in contralateral non-injured quadriceps (**B**) For **A**, *n* = 62 (Ctrl) animals over nine independent experiments at 0 dpi, *n* = 28 (Gly) and 7 (CTX) animals over four independent experiments at 1 dpi, *n* = 3 (Gly and CTX) animals over three independent experiments at 3, 7 and 9 dpi. For B, *n* = 62 (Ctrl), 19 (Gly), 6 (CTX) animals over 9, 3, and 3 independent experiments, respectively. **C** Detection of in vivo Edu incorporation detected by flow cytometry in FAPs of control and injured animals (Gly, 1 dpi). *n* = 8 animals at all time points over three independent experiments. **D** Representative confocal images and immunohistological analysis of injured (Gly and CTX) quadriceps at 1 dpi and quantification of Sca-1^+^/Podoplanin^+^/CD45^−^ cells in situ. *n* = 4 (Ctrl and Gly) and 5 (CTX) animals over three independent experiments. Bar scale 50 μm. **E**, **F** Clonogenic (**E**) and adipogenic (**F**) assays were performed on total SVF isolated from control or injured (Gly and CTX) muscle at 1 dpi. For E, *n* = 12 (Ctrl) and 5 (Gly and CTX) animals over three independent experiments. **F** *n* = 8 (Ctrl) and 14 (Gly), and 6 (CTX) animals over four independent experiments. **G** Representative phase contrast images of Ctrl, Gly, or CTX muscle-derived SVF cells under adipogenic culture conditions. Cells were fixed at day 4 and stained with Oil red O. Bar scale 50 μm. **H** mRNA expression of adipogenic markers measured on total SVF isolated from control or injured (Gly and CTX) muscle at 1 dpi. *n* = 7 (Gly and CTX) animals over four independent experiments. Results are expressed as a percentage of non-injured control animals with mean ± SEM; *p < 0.05, **p < 0.01, ***p < 0.001, ****p < 0.0001 vs Ctrl.

To investigate whether migrating ASCs may account for the pool of FAPs in the early steps following muscle injury, we first analyzed the number of FAPs in the quadriceps muscle 24 h following damage using glycerol (Gly) and myotoxin (cardiotoxin, CTX). Both Gly- and CTX-induced damages are well-characterized murine models of muscle injury at the functional, histological and inflammatory levels (Figures S1B, C)^31,32^. The cell composition of the whole muscle-derived stroma-vascular fraction (SVF) was analyzed by flow cytometry and FAPs content (identified as Sca-1^+^/CD34^+^/CD140α^+^/CD31^−^/CD45^−^ cells was measured from 1 to 9 dpi (Fig. 2A). As previously reported^4,5,33^ and with either muscle-injury model, muscle FAP number peaked at 3 dpi and returned to basal value at 9 dpi (Fig. 2A). Also in accordance with previous studies, FAP number drastically increased by 1 dpi in the injured muscle (>5-fold, Fig. 2A, without being affected in the contralateral non-injured side, Fig. 2B). The rise in FAPs at 1 dpi was not due to their proliferation, since no differences in EdU (a nucleotide analog) incorporation between FAPs from injured and control mice were detected (Figs. 2C, S1D), whereas other cell population, such as leukocytes, start proliferating by 1 dpi (Figure S1E). The FAP increase evidenced by flow cytometry at 1 dpi was then confirmed in situ, by immuhistochemistry of the damaged muscle (Fig. 2D). In accordance with our transcriptomic results, the majority of FAP cells were podoplanin^+^ at 1 dpi (Fig. 2D). To functionally demonstrate the presence of increased FAP number at 1 dpi, we tested colony-forming unit-fibroblast (CFU-f) activity of muscle-derived SVF from injured (Gly or CTX) or non-injured animals at 1 dpi. As expected, the early rise in FAP number following muscle injury was associated with a rise in the CFU-f activity (Fig. 2E). Moreover, given that FAPs are the only cell type expressing adipogenic potential in the muscle^5,25,33,34^, in vitro adipogenesis of whole muscle-derived SVF from control or 1 dpi animals was tested. Upon adipogenic induction, muscle-derived SVF originating from injured animals clearly accumulated more lipids and expressed higher levels of adipogenic markers (Figs. 2F, G, H) confirming the rise in FAP content in the injured muscle at 1 dpi.

**Fig. 3:**
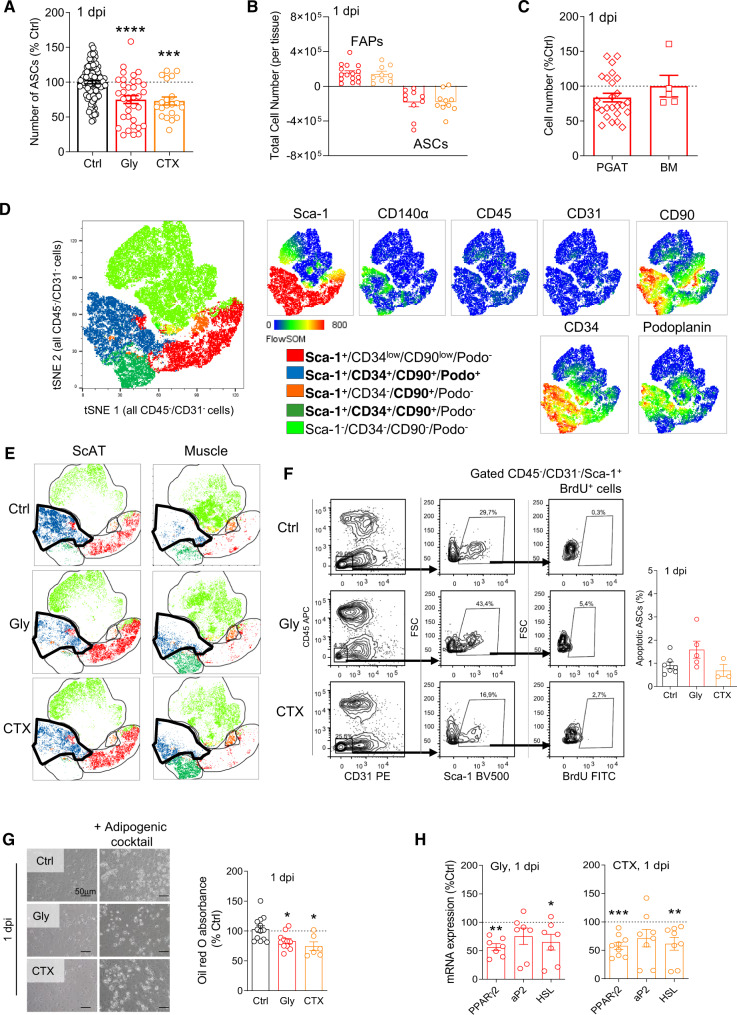
ASC content diminishes in ScAT within 24 hours after muscle injury. **A** Flow cytometry analyses of CD31, CD45, Sca-1, CD34, and podoplanin expression in ScAT-derived SVF of control and injured (Gly and CTX) animals at 1 dpi. *n* = 106 (Ctrl), 34 (Gly), and 19 (CTX) animals over 15, 15, and 9 independent experiments respectively. **B** Relative cell numbers in injured muscle and ScAT at 1 dpi. *n* = 15 (Gly) and 9 (CTX) animals for muscle and 10 (Gly) and 10 (CTX) animals for ScAT over three independent experiments. **C** ASCs and MSCs content in perigonadic adipose tissue (PGAT) and bone marrow (BM), respectively, using flow cytometry at 1 dpi in Gly-injured animals. *n* = 25 (PGAT) and 5 (BM) animals over three independent experiments. **D** ASCs and FAPs phenotypic analysis by flow cytometry. Merged tSNE plot for all control and Gly-injured CD45^−^/CD31^−^ cell among ScAT and muscle SVF-derived cells. The identity of each cluster according to the combinatory expression level of multiple markers is color-coded in the tSNE plot. Cytometry marker expression level plots are presented on the right-hand side. **E** Comparison of ScAT and muscle tSNE plot in control and injured (Gly and CTX) conditions at 1 dpi; clusters identified in **D** are black circled. **F** TUNEL staining of ScAT-derived SVF from Gly- or CTX-injured, or non-injured animals (Ctrl) at 1 dpi. *n* = 7 (Ctrl), 5 (Gly), and 3 (CTX) animals over three independent experiments. **G** Phase contrast images of adipogenic challenged ScAT-derived SVF from Ctrl, Gly, and CTX-injured animals. Cells were fixed at day 4 of differentiation and stained with oil red O. Bar scale 50 μm. *n* = 13 (Ctrl), 11 (Gly), and 9 (CTX) animals over four independent experiments. **H** mRNA expression of adipogenic genes in ScAT-derived SVF from Ctrl, Gly, and CTX-injured animals at 1 dpi. *n* = 7 (Ctrl), 7 (Gly), and 7 (CTX) animals over three independent experiments. Results are expressed as percentage of non-injured control animals with mean ± SEM; *p < 0.05, **p < 0.01,***p < 0.001 vs Ctrl.

In parallel, we studied ASCs content in ScAT following muscle injury. ASCs were identified as Sca-1^+^/CD34^+^/CD31^−^/CD45^−^ cells as previously reported^18,19^. Concomitant with the increase in FAP number in the injured muscle, the number of ASCs significantly decreased by 20–25% in the ScAT at 1 dpi (Fig. 3A), both following Gly or CTX damage. Importantly, the gain in muscle FAP number was found equivalent to the loss in ScAT-ASCs, whatever the injury model used (Fig. 3B). To verify that muscle injury specifically triggered the decrease in ASCs, and to rule out any impact of the injury procedure on the cell content of ScAT, NaCl was injected into the quadriceps muscle. NaCl neither induced muscle damage (Figure S2A, B) nor modified ASC and/or leukocyte content in ScAT (Figures S2C, D), demonstrating that muscle injury per se triggers the fall in ASCs content observed in the ScAT at 1 dpi. Moreover, the ScAT was specifically impacted by muscle damage since neither perigonadic AT (PGAT) nor the bone marrow (BM), another tissue source of MSCs, were affected at 1 dpi (Fig. 3C). Whether all ASCs or subpopulations of ASCs were injury-responsive was then addressed. Based on our transcriptomic analysis, extensive flow cytometry analysis of AT and muscle-derived freshly harvested SVF originating from injured or non-injured animals was performed. We used the dimensionality reduction algorithm, t-distributed stochastic neighbor embedding (tSNE), to qualitatively assess cell population diversity and heterogeneity^35,36^ in the CD45^−^/CD31^−^ cell subset. In contrast to conventional sequential biaxial plot-based analysis, tSNE analysis generated a single map in which the complex multi-dimensional geometric relationships between single cells were represented in a two-dimensional space. In the CD45^−^/CD31^−^ cell populations, five distinct cell subsets were deciphered on the tSNE map based on the expression of Sca-1, CD34, CD90, CD140α and podoplanin (Fig. 3D). Next, we investigated the impact of muscle injury (Gly and CTX, 1 dpi) on the proportion of these five cell subsets in both the muscle and the ScAT. Among the five cell subsets identified by tSNE analysis, the Sca-1^+^/CD34^+^/CD90^+^/Podoplanin^+^/CD31^−^/CD45^−^ cell population was increased in the injured muscle while it disappeared in the ScAT at 1 dpi (Gly or CTX) (Fig. 3E).

To examine whether programmed cell death could be responsible for the drop in ASCs content in the ScAT, we measured DNA fragmentation (TUNEL) after Gly or CTX muscle damage at 1 dpi. Frequencies of TUNEL-positive ASCs were not modified in the ScAT (Fig. 3F). As for the muscle, ASCs are the only cell population in AT-derived SVF capable of adipogenic potential^19,37^. Therefore, in vitro adipogenesis of whole ScAT-derived SVF from control or injured animals was tested and compared (Fig. 3G) to functionally assess ASC content. In agreement with flow cytometry results, the drop in ASCs content was associated with a decrease in lipid accumulation and expression of adipogenic markers in ScAT-derived SVF originating from injured animals (Fig. 3H). To rule out putative effects of CTX or Gly on the metabolic status of the animals, and thus on the biology of AT, body weight and plasmatic glucose levels of the animals was monitored during 28 days following muscle injury. Neither the body weights of the animals nor their glycaemia was modified by muscle lesion (Figure S2E, F).

Collectively our data show that the early rise in FAPs content following muscle injury is associated with a decline in ASCs content and that Podoplanin may be a selective marker of this cell population.

### Muscle injury or exercise triggers ASC chemotaxis in humans and mice

**Fig. 4:**
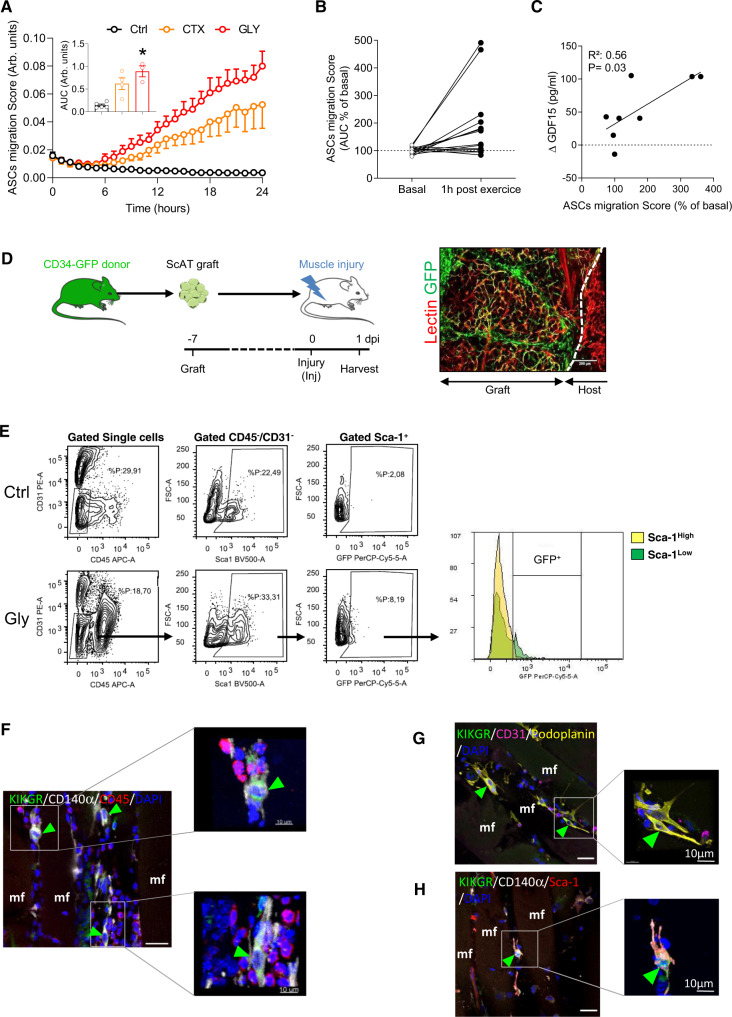
ASCs leave the ScAT and infiltrate injured muscle. **A** Time course evaluation of murine in vitro ASC chemotaxis in response to plasma isolated from Ctrl, CTX- and Gly-injured animals at 1 dpi. *n* = 5 (Ctrl), 3 (Gly) and 4 (CTX) animals over three independent experiments. **B** In vitro human ASCs (of three individuals) chemotaxis in response to serum of six individuals collected 1 hour after an acute bout of continuous exercise (60% VO_2_ max). **C** Correlation of human ASC chemotaxis with 1h-post exercise GDF-15 blood levels. *n* = 6 serums tested on 1 or 2 sets of ASC over three independent experiments. **D** Model of ScAT grafting from CD34-GFP mouse into WT C57Bl/6 mice (left panel), the figure was partly generated using Servier Medical Art, provided by Servier, licensed under a Creative Commons Attribution 3.0 unported license. Immunohistofluorescence image of the ScAT depot 7 days post-graft surgery (right panel, bar scale 200 μm). **E** Flow cytometry analysis of the SVF from Gly-injured muscle of the grafted mice (1 dpi), GFP^+^/CD45^−^/CD31^−^ are scated on an histogram for Sca-1 intensity. **F**–**H** Immunohistological analysis of Gly-injured (1 dpi) quadriceps in grafted mice with KikGR ScAT in situ (green arrowheads point KikGR^+^/CD140α^+^/CD45^−^ (**F**), KikGR^+^/ Podoplanin^+^/CD31^−^ cells (**G**) or KikGR^+^/CD140α^+^/Sca-1^+^ (**H**)). Bar scale 10 μm. Results are expressed as mean ± SEM; *p < 0.05.

Given these results, we hypothesized that the drop in ASCs in response to muscle injury resulted from their egress from the ScAT. Consequently, we investigated in vitro whether serum from injured animals could trigger ASCs chemotaxis. We collected serum from control or injured (1 dpi) animals and ASCs chemotaxis was studied and compared. We found that whatever the injury model, serum from injured animals strongly induced the chemotaxis of ASCs (Fig. 4A) supporting the hypothesis that muscle injury may trigger the egress of ASCs from ScAT via the production of blood circulating factors. We also investigated whether such a mechanism may occur in humans. To do so, we took advantage of blood samples originating from a clinical study investigating the impact of an acute bout of continuous exercise^38^ in young, active men (Table 1) as a putative source of muscle damage and/or fatigue. As we measured in mice, the chemotactic response of human-native ASCs towards the serum of each individual before and after exercise was studied. As shown in Fig. 4B, the chemotactic response of ASCs toward serum at post-exercise was increased by two- to fivefold compared with the pre-exercise condition, though heterogeneity was observed from one individual to another. We then wondered whether such a disparity could be explained by inter-individual variability to exercise-mediated stress response. Growth differentiation factor 15 (GDF-15) is induced under stress conditions, to maintain cell and tissue homeostasis^39^. Recent studies also suggested that GDF-15 is an exerkine^38^, exhibiting a possible protective role in exercise-induced muscle injury or inflammation^40–43^. Therefore, circulating blood levels of GDF-15 were measured before and after exercise to assess the exercise-induced stress response intensity of each individual (Table 2). In agreement with our hypothesis and the results obtained in mice, the chemotactic activity of ASCs is strongly correlated with GDF-15 levels akin to an index of muscle fatigue (Fig. 4C). These findings indicate that blood factors released after muscle injury/stress may trigger the mobilization of ASCs from the ScAT, in both mice and humans.

**Table 1:** Anthropometry and body composition of the individuals enrolled in the clinical study

Subject ID	Age	Weight (kg)	Height (cm)	BMI (kg/m²)	% Fat Mass	Fat mass (kg)	Free fat mass (kg)
A	22	79	184	23.33	12.16	8.5	62.1
B	19	87	193	23.36	17	13.6	63.9
D	19	70	171	23.94	17.8	11.1	49.4
I	27	87.2	185	24,54	15.9	12.8	64.8
J	24	79.3	191	21.7	15.6	10.6	60.7
M	29	71.3	176	23.01	14.3	10.4	59.2

**Table 2:** GDF-15 blood levels in individuals before (pre) and after (post) an exercise on a bicycle ergometer at 60% VO2 peak for 1-hr

GDF-15 (pg/ml)			
Subject ID	Pre	Post	Δ (Post - Pre)
A	153,4	168,1	14,7
B	417,0	457,5	40,5
D	209,4	313,2	103,8
I	341,2	446,5	105,3
J	451,6	437,7	−14,0
M	263,8	329,5	65,6

Δ corresponds to the difference in GDF-15 blood level, before, and after the exercise.

### ASCs exit the ScAT and infiltrate the muscle after injury

To determine whether ASCs can leave the ScAT in response to muscle injury to further infiltrate it, we performed in vivo experiments in mice. However, due to the absence of unique specific ASC marker, no mouse model is so far available to visualize, in vivo, the trafficking of native ASCs from AT to any other tissue compartments. To overcome this obstacle, we first used Tg(Cd34- EGFP)MF6Gsat/Mmucd (referred to as CD34-GFP, an ASC surface marker) from which a piece of ScAT was grafted into the ScAT of a non-GFP recipient mouse (Fig. 4D). Fat graft revascularization, an index of viability, was verified by using retro-orbital injection of Rhodamin-lectin into grafted animals (Fig. 4D, S3A). At day 7 post-graft, muscle was damaged and the presence of GFP^+^ cells into the injured muscles was assessed at 1 dpi. CD31^−^/CD45^−^/Sca-1^+^/GFP^+^ cells were identified in injured muscles by flow cytometry (~8% of CD31^−^/CD45^−^/Sca-1^+^ cells, Fig. 4E) while by contrast no GFP cells were detected either in the muscle of a CD34-GFP ScAT grafted non-injured control animal (Fig. 4E), nor in a non-grafted mouse (Figure S3B). Recently, Giuliani et al.^44–46^ reported that Sca-1 expression defines two FAPs subsets, the Sca-1^High^ and Sca-1^low^ FAP subpopulations, and that Sca-1 level expression mirrors FAPs biological fate. Consequently, we analyzed Sca-1 level expression in muscle-infiltrated ASCs and found that GFP^+^-ASCs were mainly in the Sca-1^low^ subpopulation (Fig. 4E). To verify GFP expression specificity, FAPs from grafted injured (1 dpi) or non-injured animals were sorted and genomic GFP expression was analyzed. Genomic GFP expression was found in FAPs of injured muscles (Figure S3C), in contrast to control muscles from grafted animals (Figure S3C) or other distant organs (Figure S3D). To rule out possible mouse model effect, ScAT graft from other fluorescent transgenic mouse models the Rosa26^mT/mG^ or the *CAG::KikGR*^33^ animals^47,48^ were used (Figure S3E). Here again, we observed in mice grafted with the Rosa26^mT/mG^ that ~10% of Tomato^+^-cells among the CD45^−^/CD31^−^/Sca-1^+^ cells were found in the injured muscle, corresponding to infiltrated ASCs originating from the red fluorescent mT/mG graft pad (Figure S3F). Interestingly, the Tomato^+^-cells laid preferentially among the CD45^−^/CD31^−^/Sca-1^low^ cell subpopulation (Figure S3F, lower panel) such as observed previously in Fig. 4E. Collectively, our result demonstrates that following muscle injury, ScAT releases FAP-like cells, which specifically infiltrate the damaged muscle. Such specific post-damage cell infiltration originating from AT may largely contribute to the heterogeneity in Sca-1 expression recently described in the FAPs and which may impact the fate decision of FAPs^46^. Further analysis of their immunophenotype in vivo was performed using either CD34-GFP or KikGR AT grafted animals at 1 dpi. The infiltrated fluorescent cells were found to be Sca-1^+^/CD34^+^/Podoplanin^+^/CD140α^+^/CD31^−^/CD45^−^, corresponding to the ASCs immunophenotype (Fig. 4F–H, S3G). Altogether, these findings show that the first wave of FAP increase following muscle injury at 1 dpi largely results from ASC mobilization and infiltration into the damaged muscle.

### ASC muscle infiltration involves platelets

**Fig. 5:**
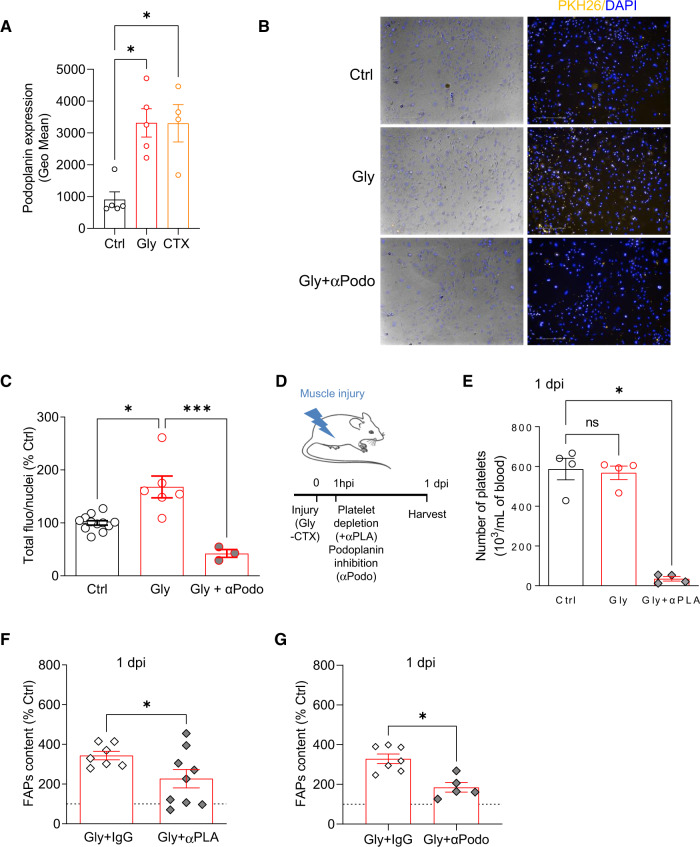
The interaction of blood platelets with ASCs determines their infiltration into the damaged muscle. **A** Cell surface podoplanin expression of ScAT-derived ASCs of injured (Gly and CTX) or control animals by flow cytometry at 1 dpi. *n* = 5 (Ctrl), 5 (Gly), and 4 (CTX) animals over three independent experiments. **B** Representative phase contrast and fluorescent images of PKH26-stained platelets (yellow) co-incubated with ScAT-ASCs in the presence or not of blocking podoplanin antibody, bar scale 200 μm. **C** Quantification of PKH26-stained platelets with ASCs. *n* = 12 (Ctrl), 6 (Gly), and 3 (Gly + αPodo) animals over three independent experiments. **D** Model of platelet depletion, the figure was partly generated using Servier Medical Art, provided by Servier, licensed under a Creative Commons Attribution 3.0 unported license. **E** Platelet numeration 1 day post platelet depletion. *n* = 4 (Ctrl), 4 (Gly), and 4 (CTX) animals over two independent experiments. **F** FAPs content by flow cytometry in injured muscle from control (+IgG) or platelet-depleted animals (+αPLA). *n* = 7 (Gly+IgG) and 9 (Gly + αPLA) animals over two independent experiments. **G** FAPs content by flow cytometry in injured muscle from control (+IgG) or antipodoplanin treated animals (+αPodo). *n* = 7 (Gly+IgG) and 5 (Gly + αPodo) animals over three independent experiments. Results are expressed as percentage of non-injured control animals with mean ± SEM; *p < 0.05, **p < 0.01, ***p < 0.001.

Our current understanding of in vivo trafficking of native ASCs/MSCs is still very incomplete^49^ and mostly derives from in vitro experiments. However, some studies indicate that platelets control MSC trafficking and/or recruitment to sites of injury^50–53^. We found that the mobilizable ASC cell subset was podoplanin^+^ and observed an upregulation of podoplanin expression at the cell surface of ASCs from injured animals measured by flow cytometry (Fig. 5A). Podoplanin is an endogenous ligand for C-type lectin-like receptor 2 (CLEC-2) which is an essential platelet-activating receptor. Consequently, we next assessed whether ASC and platelet interaction are necessary for the recruitment of ASCs to the injured muscle. We used an in vitro assay whereby platelets, isolated from control or injured animals (1 dpi) were fluorescently labeled and co-incubated with ASCs (isolated from ScAT). The adhesion of platelets to ASCs was evaluated and compared. The results show that platelets originating from injured animals adhered more to ASCs in vitro (Fig. 5B, C), a physical interaction that was completely abolished when an antibody directed against podoplanin was added (Fig. 5B, C). Given our results showing that both ASC mobilization and infiltration account for the first wave of FAP rise in injured muscle and that platelets originating from injured animals interact more with ASCs, we next investigated the consequences of platelet depletion in vivo on FAP augmentation following muscle injury. Muscle damage was induced as described earlier and either platelet depletion or podoplanin inhibition was performed 1 h later (Fig. 5D, E). FAP content was then quantified by flow cytometry at 1 dpi. Both platelet depletion and podoplanin inhibition diminished the increase of FAPs in the muscle by more than twofold at 1 dpi (Fig. 5F, G) suggesting that the infiltration of ASCs into the injured muscle involves platelets.

### ASC muscle infiltration is needed for effective regeneration

**Fig. 6:**
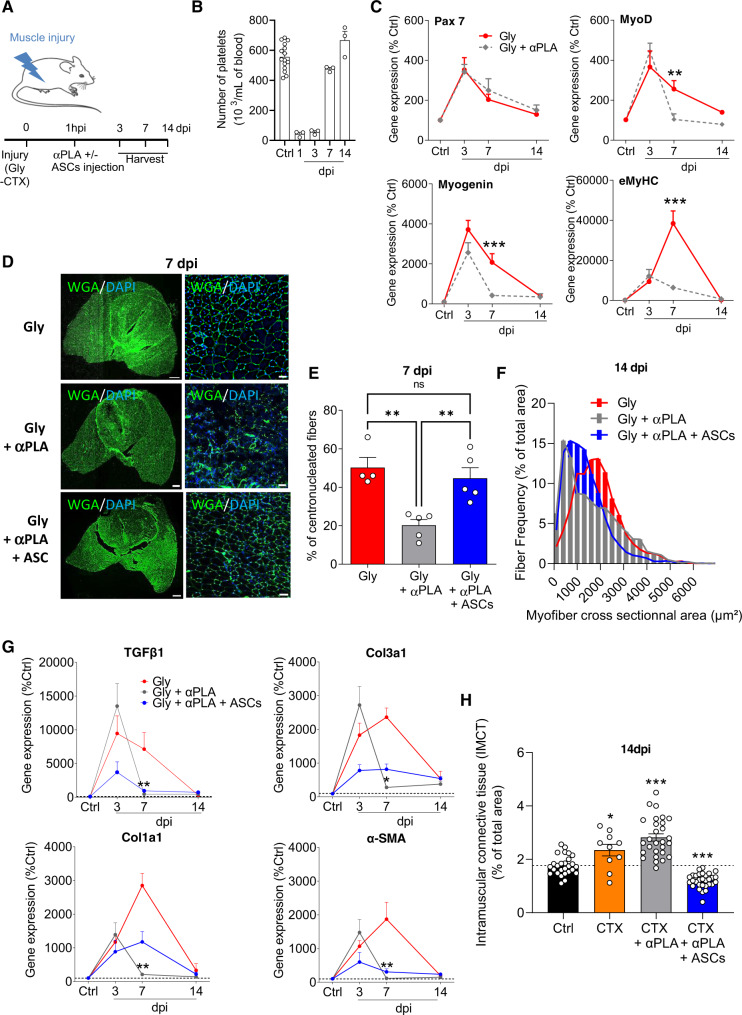
The interaction of blood platelets with ASCs is crucial for effective muscle regeneration. **A** Model of platelet depletion and ASCs injection. The figure was partly generated using Servier Medical Art, provided by Servier, licensed under a Creative Commons Attribution 3.0 unported license. **B** Platelet numeration time course in control and Gly-injured animals from 1 to 14 dpi. *n* = 15 (Ctrl) and three animals for each other time point over two independent experiments. **C** Time course of mRNA expression of myogenic genes in quadriceps muscle from control and Gly-injured animals with and without platelet depletion. *n* = 12–16 (Ctrl) and 5–8 at 3 dpi, 6–8 at 7 dpi, and 6–9 at 14 dpi animals over four independent experiments. **D** Immunohistological confocal images of quadriceps muscles at 7 dpi (Gly +/− platelet depletion). Bar scales 500 (left) and 50 (right) μm. **E** Immunohistological-based quantification of regenerative (WGA centronucleated fibers) muscle fibers at 7 dpi. n = 5 (Gly), 5 (Gly + αPLA) and 4 (Gly + αPLA + ASCs) animals over four independent experiments. **F** Size distribution of regenerating muscle fibers in Gly-injured (red), platelet-depleted (gray), and ASCs supplemented (green) animals at 14 dpi. *n* = 4 (Gly), 6 (Gly + αPLA), and 5 (Gly + αPLA + ASCs) animals over four independent experiments. **G** Time course of mRNA expression of intramuscular connective tissue (IMCT) genes in quadriceps muscle from control and Gly-injured animals with and without platelet depletion. *n* = 5 (Ctrl) and, 5–7 at 3 dpi, 3–4 at 7 dpi, 4 at 14 dpi animals over three independent experiments. **H** Immunohistological-based quantification of IMCT deposition in quadriceps muscle from control and CTX-injured animals with and without platelet depletion at 14 dpi. *n* = 27 (Ctrl), 10 (CTX), 28 (CTX + αPLA), and 30 (CTX + αPLA + ASCs) pictures of 4, 3, 4, and 4 animals in each group, respectively, over three independent experiments. Results are expressed as a percentage of control animals with mean ± SEM; *p < 0.05, **p < 0.01, ***p < 0.001.

We next studied the impact of FAPs rise disruption on the muscle regeneration process. To do so, we further exploited the muscle injury model with platelet depletion until 14 dpi (Fig. 6A). Platelets depletion was maximal at 1 dpi, during the time of ASCs infiltration, before going back to basal in the later days, avoiding side effects due to long-term imbalance (Fig. 6B). The expression of genes involved in the molecular program of muscle regeneration was studied following muscle injury until 14 dpi. Even though the expression patterns of early genetic markers of the muscle regeneration program (*pax7*, *myf5*, *mrf4*) were not modified (Fig. 6C, S4), the expression of late myogenic markers (*myod*, *myog*, *myHC-emb*) were clearly modified in platelet-depleted animals compared with controls (Fig. 6C). To reinforce these results, we quantified both the number of newly synthesized centrally nucleated fibers and the fiber diameters, as markers of the regenerative process and found that the proportion of centrally nucleated fibers at 7 dpi as well as the cross-sectional area distribution of regenerating fibers at 14 dpi were dramatically diminished in platelet-depleted animals (Fig. 6D–F). In accordance with our hypothesis, the intramuscular injection of ASCs rescued the tested parameters (Fig. 6D–F). Beyond the formation of new myofibers, muscle regeneration also requires connective tissue restoration, via the secretion and organization of newly synthetized extracellular matrix^54^. We thus investigated the gene expression time course involved in this process up to 14 dpi (Gly) with initial platelet depletion. All the four markers studied (*tgfb1, col3a1, col1a1, and -sma*) followed a dynamic expression profile upon glycerol injury that was disturbed upon platelet depletion. With ASCs injection such dynamic was partially restored though in a variable way from gene to gene (Fig. 6G). On the other hand, excessive and persistent intramuscular connective tissue deposition (IMCT) is a hallmark of fibrosis^55,56^. Knowing that the CTX injury model is described to be more pro-fibrotic compared to the Gly one, we investigated the consequence of platelet depletion in this context^57^. At 14 dpi, CTX injury causes a significant IMCT deposition that is worsened upon platelet depletion (Fig. 6H). Conversely, ASCs intramuscular injection improved IMCT levels, possibly indicating a pro-regenerative role following muscle injury (Fig. 6H).

### ASCs’ tissue origin determines muscle regeneration outcome

**Fig. 7:**
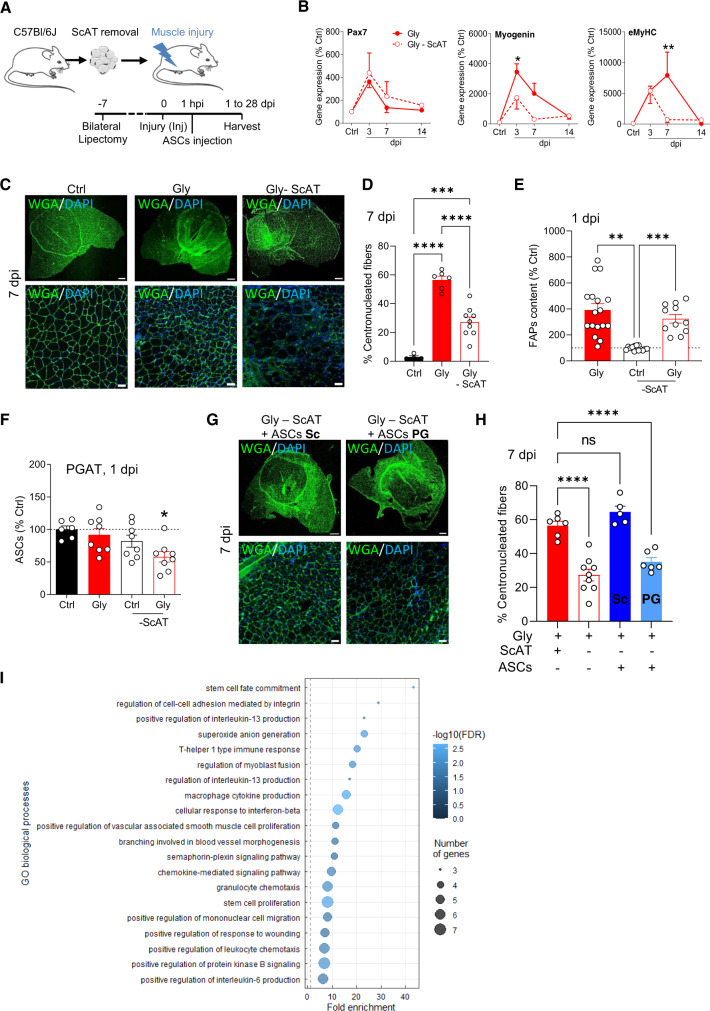
The origin of the ASCs reservoir determines muscle regeneration outcome. **A** Model of bilateral lipectomy and ASC injection. The figure was partly generated using Servier Medical Art, provided by Servier, licensed under a Creative Commons Attribution 3.0 unported license. **B** Time course of mRNA expression of myogenic genes in quadriceps muscle from control and Gly-injured animals with and without lipectomy. *n* = 4 (Ctrl) and 3–4 at 3 dpi, 3–4 at 7 dpi, 3–4 at 14 dpi animals over three independent experiments. **C** Immunohistological confocal images of quadriceps muscles at 7 dpi (Gly +/− platelet depletion). Bar scales 500 (top) and 50 (bottom) μm. **D** Immunohistological-based quantification of regenerative (WGA centronucleated fibers) muscle fibers at 7 dpi.n = 4 (Ctrl), 6 (Gly), and 9 (Gly-ScAT) animals over three independent experiments. **E** FAP number quantification in Gly-injured or control muscle with or without ScAT lipectomy. *n* = 17 (Gly), 13 (Ctrl-ScAT), and 11 (Gly-ScAT) animals over five independent experiments. **F** ASC number quantification in Gly-injured or control PGAT with or without ScAT lipectomy. *n* = 6 (Ctrl), 8 (Gly), 8 (Ctrl-ScAT), and 8 (Gly-ScAT) animals over four independent experiments. **G** Immunohistological confocal images of quadriceps Gly-injured and control muscles at 7 dpi with or without lipectomy. Bar scales 500 (top) and 50 (bottom) μm. **H** Immunohistological-based quantification of regenerative (WGA centronucleated fibers) muscle fibers at 7 dpi. *n* = 6 (Gly), 9 (Gly-ScAT), 6 (Gly-ScAT+ASC(ScAT)), and 5 (Gly-ScAT+ASC(PGAT)) animals over five independent experiments. **I** Go term analysis between ScAT and PGAT-derived ASCs. Results are expressed as percentage of control animals with mean ± SEM; *p < 0.05, **p < 0.01, ***p < 0.001, ****p < 0.0001 vs Ctrl.

To further evaluate the impact of ASC infiltration on the muscle regeneration process, ScAT from both sides of the animal were removed to eliminate the injury-responsive reservoir of ASCs (Fig. 7A). We first verified the impact of bilateral lipectomy on both morphometric and metabolic parameters. Body weight and total fat mass were not significantly modified (Figure S5A, B). PGAT, the other main adipose depot, did not overgrow to compensate the removal of ScAT (Figure S5C). Food intake was only affected the first days after the surgery but normalized on later time points (Figure S5D). Finally, blood glucose was checked and did not show significant changes after the bilateral lipectomy (Figure S5E). Muscle injury was then performed as described earlier. It did not affect the weight of the remaining PGAT and the injured muscle (Figure S5F, G). The expression of genes involved in the molecular program of muscle regeneration was then examined. As for platelet-depleted animals, the expression pattern of *pax7* was not modified (Fig. 7B) while the one of both late myogenic markers *myog*, and *myhc-emb*, was clearly affected (Fig. 7B). Accordingly, the number of newly synthesized centrally nucleated fibers, was diminished in ScAT-depleted animals (Fig. 7C, D). To our surprise, in ScAT-depleted animals, the early rise in FAPs content still occurred, in contrast to our prediction (Fig. 7E). Unlike the control animals, we found that the ASCs content of PGAT was diminished at 1 dpi when ScAT was removed (Fig. 7F). Consequently, these data suggest that in the absence of ScAT, PGAT depot takes over in terms of infiltration of FAP-like cells, yet without an efficient muscle regeneration outcome. This suggests that according to their fat depot source, ASCs may not exhibit similar regenerative potential. We thus compared the impact of ScAT- or PGAT- ASCs on muscle regeneration outcome in the context of ScAT reservoir removal. In ScAT-depleted animals the injection of ScAT-derived ASCs entirely rescued the level of centronucleated fibers while the ones from PGAT did not (Fig. 7G, H). Finally, we wondered whether ScAT-derived ASCs harbor a specific pro-regenerative profile that promotes better muscle regeneration. Functional analysis by gene ontology (GO) revealed specific gene expression profiles in ScAT-ASCs when compared to PGAT-ASCs. Indeed, there was an enrichment in ScAT-ASCs in the biological functions related to coordination of inflammation, stem cell proliferation and fate, and myoblast fusion (Fig. 7I, Supplementary data 1), all together being essential pathways to allow muscle regeneration in response to acute injury.

## Discussion

The regenerative capacity of skeletal muscle mostly relies on satellite cells (SCs), which proliferate in response to exercise or following myotrauma, to repair the injured muscle^3,58^. However, in the past decade many other cell types have been shown to contribute to this process in order to maintain skeletal muscle integrity and functions^59,60^.

Among those diverse and heterogeneous cell types, resident mesenchymal progenitors named FAPs have emerged as key players in skeletal muscle regeneration and disease by providing functional support to SCs to perform efficient muscle regeneration^7,61,62^. Upon muscle injury, FAPs become activated and expand rapidly^4,5^. Indeed, the number of FAPs peaks 96 h post-muscle damage^9^ together with their proliferative activity^4^. Surprisingly, before FAPs proliferation is fully activated^4,5,8,63^, FAPs number dramatically increases within the 24 h following muscle injury and the origin of such rise has never been addressed in the literature so far^8,9,15,64^. Furthermore, various recent studies addressed the question of muscle resident cell heterogeneity in homeostasis and regenerative conditions using single-cell analysis^15,17,60,65^. In that context, Malecova et al^15^ identified the appearance of a subpopulation of Tie2^low^FAPs within the 24 h following muscle injury in a mouse model, which they considered a reflection of the cell state of FAPs during muscle regeneration^15^.

The results presented here provide a mechanism that may explain both the rapid FAPs number increase observed 24 h after muscle injury without proliferation and FAPs heterogeneity. We show that in response to acute muscle injury ASCs egress ScAT and infiltrate the damaged muscle, a mechanism necessary for efficient muscle regeneration. Moreover, we propose that a subpopulation of mesenchymal progenitors originating from ScAT participates to the pool of FAPs and could correspond to the subpopulation of “activated” FAPs reported by Oprescu et al^17^. Based on our results it seems likely that this FAP subpopulation originates from ScAT rather than being a transitory cell state of resident FAPs. Our data also suggest that such a mechanism may occur in humans in response to exercise-induced muscle fatigue. Indeed, GDF-15 has been implicated in exercise and exercise recovery and that high levels of GDF-15 may signal exercise aversion to prevent injurious behavior^66^. We show here that human ASCs migration score positively correlates with exercise-mediated GDF-15 level increase^41^, which may indicate that ASCs are mobilized when muscle signals a pre-injury state.

The mechanisms controlling the trafficking of endogenous MSCs and/or ASCs are poorly understood and our current understanding mostly derives from in vitro studies^49^. We show here that in response to muscle injury, ASCs within ScAT overexpress podoplanin, which is a ligand for the platelet receptor CLEC-2 (for C-type lectin-like receptor 2)^67^. In agreement with this result, several studies report that the majority of infused MSCs can be found within the circulation in close contact with platelets^50,51^ to facilitate their homing to inflamed tissues^30,68^. Our data show that platelets originating from muscle- injured animals adhere more in vitro to ASCs and that neutralizing the CLEC-2/podoplanin interaction completely abolished this effect, suggesting a role for platelets in ASCs trafficking in vivo. This was confirmed in platelet-depleted animals where FAPs increase was strongly inhibited in response to muscle injury. The blunting in FAPs increase, observed in platelet-depleted animals was associated with impaired muscle regeneration that was rescued by intramuscular ASC injection. The precise mechanisms by which platelets control ASC egress and/or muscle infiltration are still unclear and need to be addressed in further studies. However, platelets have been shown to maintain endothelial permeability while the trafficking of infused MSCs to inflamed sites is facilitated by increased endothelial permeability^65^. Moreover, recent studies demonstrate that podoplanin expression imparts a pro-migratory phenotype in MSCs, facilitating their migration across the vessel wall and interaction with circulating platelets^30^.

To rule out platelet-dependent effects mediated by their secretion on muscle regeneration we set up another experimental model by the removal of ASCs source, i.e., a bilateral ScAT lipectomy. Though muscle regeneration was also strongly affected in that context, FAPs first wave at 1 dpi was unexpectedly maintained. We showed that in absence of ScAT, another AT depot, the PGAT, was solicited. Thus suggesting that when the bone fide source of ASCs is missing, another reservoir takes over but fails to support muscle regeneration. Actually, when directly injected into the injured muscle, PGAT-derived ASCs were not able to rescue muscle regeneration unlike their ScAT counterpart. Further transcriptomic analysis showed that ScAT- vs PGAT- derived ASCs are intrinsically functionally different. Extensive literature reports that FAPs secrete numerous factors being responsible for immunomodulation and IMCT remodeling after damage that support muscle SC expansion, differentiation, self-renewal, and quiescence^11,61,62,69–71^. Our transcriptomic analysis of both ASC subpopulations indeed confirmed that they exhibit profound differences regarding those essential parameters for optimal muscle regeneration. It is well accepted that visceral AT and ScAT are functionally and metabolically distinct, the latter being more “protective”^72^. The present data provide a new set of evidence that ScAT exhibits another protective function during skeletal muscle regeneration. Our data reinforce the particular relationship between muscle and AT, mainly described until now by secretory crosstalk, by showing that cell exchanges between these two organs take place and impact their homeostasis^73^.

In conclusion, our work identifies an unsuspected role of ScAT in skeletal muscle regeneration. These findings introduce the concept of AT as an endogenous supplier of regenerative cells allowing skeletal muscle to regenerate efficiently. Whether such dialog is decisive in pathological contexts or during ageing where the repartition of adipose sources is dramatically affected remains to be investigated.

## Methods

### Animal experimental protocols

This work was submitted to and approved by the Regional Ethic Committee and registered to the French Ministère de la Recherche. Animals were kept under controlled light (12-hr light/dark cycles; 07h00–19h00), temperature (20 °C–22 °C), hygrometry (40% ± 20%) and fed ad libitum a chow diet (8.4% fat, Safe®A04, Safe lab).

#### Muscle injuries

8–12 weeks old male C57Bl/6 J mice (Janvier) were anesthetized with isoflurane and 80 µL of 10 µM cardiotoxin (Sigma, C9759) or 50% glycerol in saline solution (NaCl 0.9%) were injected into the right quadriceps.

#### ScAT grafting

Three transgenic fluorescent mice models were used as ScAT donors: the Tg(Cd34-EGFP)MF6Gsat/Mmcd (MMRRC), the Rosa26^mT/mG^ and the *CAG::KikGR* animals respectively referred as CD34-GFP, mT/mG, and KikGR mice. Non fluorescent C57Bl/6 J-recipient mice received graft pads (10 mg) from the above models into their ScAT while sham control mice were only skin incised. After 7 days (grafted or sham) mice were injured.

#### Platelet depletion

Mice were injected intraperitoneally with platelet depleting antibody (anti-GPIb, EMFRET, #R300) in saline solution at a concentration of 2 mg/kg. Control animals were injected with mixture of non-immune rat IgG which display no cytotoxic effects on platelets in mice (EMFRET, #C301, 2 mg/kg). Platelet depletion efficiency was verified on 20 µl of total blood of the animals (from day 0 to 14) using Micros 60 (HORIBA Medical).

#### Antipodoplanin treatment

Mice were injected intraperitoneally with InVivoMAb anti-mouse Podoplanin (BioCell, #BE0236, 100 μg per mouse) in saline solution. Control animals were injected with inVivoMAb polyclonal Syrian hamster IgG (BioCell, #BE0087, 100 μg per mouse).

#### Bilateral lipectomy

Mice were anesthetized with isoflurane and a skin incision was performed above ScAT lymph node. ScAT was removed using forceps to disrupt conjunctive tissue adherences, and blood vessels located at the extremities were cauterized. Wound was sutured, and animals were monitored daily for 5–7 days. Muscle injured results were compared to non-injured animals, and lipectomy results were compared to sham (skin incision only) animals.

#### ASC muscle injection

For rescue experiments in both platelet-depleted and lipectomied animals, 250,000 to 300,000 freshly isolated ASCs (Sc or PG) were injected (G25 needle, 1 mL syringe in NaCl 0.9%, maximal volume of 80 µl) in anesthetized animals 1 hour after muscle injury.

#### Tissue collection

Blood was collected in anesthetized animals in the inferior cava vein with G25 needle and 1 mL syringe coated with PBS/Heparin (20 U/mL), and then dislocation was performed. Quadriceps muscle, subcutaneous (Sc) and perigonadic (PG) AT were either directly harvested for cell isolation or fresh frozen for RNAs extraction or genomic DNA (liver, heart, kidney and front limb).

### Murine AT- or muscle-SVF isolation

Freshly harvested AT or muscle were minced and SVF were obtained by enzymatic digestion.

PGAT and ScAT were digested with collagenase (NB4, Coger; 0.4 U/mL) and DNAse (1%, Roche) in αMEM (GIBCO) at 37 °C for 45 and 60 min, respectively, under constant agitation. After centrifugation (300 × *g*, 10 min, RT) and elimination of the floating mature adipocytes, the pellet containing the SVF was resuspended in erythrocyte lysis buffer (155 mmol/L NH4Cl; 5.7 mmol/L K2HPO4; 0.1 mmol/L EDTA, pH 7.3) to remove red blood cells. After filtration (34 µm sieve) and centrifugation (300 × *g*, 10 min, RT), SVF were resuspended in PBS/0.5% bovine serum albumin (BSA)/2 mM EDTA.

Quadriceps muscles were digested with collagenase B (0.5 U/mL, Roche) and dispase II (2.4 U/mL; Roche) in Hank’s Balanced Saline Solution (HBSS) + 2.5 mM Ca^2+^ for two rounds of 30 min at 37 °C under agitation separated by mechanical dissociation through G18 syringe. Reaction was stopped by adding a two volumes of αMEM + 10%NCS, then samples were filtered through 34 µm sieve and centrifuged (300 × *g*, 10 min, RT) to eliminate supernatant and to resuspend the SVF in PBS/0.5%BSA/2 mM EDTA.

Freshly harvested AT- or muscle-derived SVF were used for direct flow cytometry analysis or cell sorting if needed.

### Cell sorting

#### ASC or FAP isolation

ASC sorting: ScAT- or PGAT- derived SVF were depleted in CD45^+^ and CD31^+^ cells using anti-CD45-FITC (Miltenyi, 130-116-535) and anti-CD31-FITC antibodies (Miltenyi, 130-123-675) followed by anti-FITC magnetic microbeads (Miltenyi, 130-048-701) using an autoMACS® Pro Separator (MACS Cell Separation, Miltenyi Biotec SAS) according to the manufacturer’s instructions. CD45^−^/CD31^−^ cells were then positively sorted for Sca-1 with anti-Sca-1 magnetic microbeads (Miltenyi, 130-106-641). Isolated ASCs were used for RNAseq experiments, for muscle injection in platelet-depleted or lipectomied animals (see methods), or for in vitro platelet/ASCs interaction challenges (see methods). FAP sorting: muscle-derived SVF were treated as above and used for RNAseq experiments, affymetrix analysis (ctrl or injured), and DNA extraction.

#### Platelet isolation

For platelet isolation, total blood was collected (heparin tubes), centrifuged (5 min, 500 × *g*, RT) and the plasma-rich platelets was resuspended in Tyrode’s Buffer. Samples were centrifuged for two rounds (1900 × *g*, 8 min) and platelets were stained with PKH26 for 5 min at room temperature. After another centrifugation step (1900, 8 min, twice), platelets were resuspended in αMEM + 10% NCS (10^6^ platelets/mL). Platelets were used for in vitro platelet/ASC interaction challenges.

### Flow cytometry acquisition and data analysis

To analyze the cellular composition of freshly harvested SVF from ATs or muscles, they were incubated (25 minutes, 4 °C, dark) with Phycoerythrin (PE) CD31 (BD Biosciences #553373), CD45 (BD Biosciences #553081), Allophycocyanin (APC)-Podoplanin (Biolegend #127410) or CD140α (Biolegend #135908), Fluorescein isothiocyanate (FITC)-CD31 (BD Biosciences #553372), CD45 (BD Biosciences #553080), CD34 (BD Biosciences #553733) or Sca-1 (BD Biosciences #553335), V500- Sca-1 (BD Biosciences #561228) antibodies or the appropriate isotype controls (Rat Isotype PE IgG2a, BD Biosciences #553930; Mouse Isotype APC, Biolegend, #402012; Rat Isotype APC IgG2a, BD Biosciences, #553932; Rat Isotype FITC IgG2a, BD Biosciences, #554688; Rat Isotype BV500 IgG2a, BD Biosciences, #560786) all at dilution 1:100. After washing, the labeled cells were quantified on LSR Fortessa flow cytometer and analyzed using FACSDiva software v9.0.1 (BD Biosciences) and Flowlogic^TM^ software v7.2.1.

For the phenotypic characterization of ASCs and FAPs associated with T-Distributed Stochastic Neighbor Embedding (*tSNE*) representation, the SVFs were incubated with REA human anti-mouse antibodies (PerCP-Vio700-CD45, PE-CD31, FITC-CD34, PE-Vio770-Sca-1, APC-Vio770-CD140α, Miltenyi, respectively #130-110-801, #130-111-540, #130-117-775, #130-106-258, #130-125-991), classic rat anti-mouse VioBlue-CD90.2 (Miltenyi, #130-102-345), Viobility™ 405/520 Fixable Dye (Miltenyi, #130-130-404) and rat anti-mouse APC-Podoplanin (Biolegend #127410) and the appropriate isotype controls (Isotype PerCP-Vio700, Miltenyi, #130-113-453; Isotype PE, Miltenyi, # 130-113-450; Isotype FITC, Miltenyi, #130-113-449; Isotype PE-Vio770, Miltenyi, #130-113-452; Isotype APC-Vio770, Miltenyi, #130-113-447; Rat Isotype VioBlue IgG2b, Miltenyi, #130-102-661) all at dilution 1:100. The cells were quantified on LSR Fortessa flow cytometer and analyzed using Flowjo^TM^ software v10.6, using the tSNE tool.

### Proliferation and cell death tests

Proliferation in vivo was assessed with the Click Plus EdU 488 Flow Kit (Life Technologies) following the manufacturer’s instructions. Briefly, animals were injected IP with Edu (40 mg/g) just after the muscle injury, (i.e., 24 hr before sacrifice, such as described by Lemos et al^9^). Isolated muscle SVF-derived cells were fixed and permeabilized and further incubated for 30 min with a Click It reaction cocktail to reveal Edu staining with Alexa Fluor 488 in proliferative cells.

For apoptose/necrose evaluation, In Situ Cell Death Detection Kit (ROCHE) was used following manufacturers’ instructions. Shortly, isolated SVF-derived cells were fixed and permeabilized before the TUNEL staining (labeling solution + enzyme) for 1 hour at 37 °C. Stained cells were quantified by flow cytometry on LSR Fortessa (BD Biosciences) and analyzed using FACSDiva v9.0.1 (BD Biosciences) and Flowlogic^TM^ softwares v7.2.1.

### Clonogenic assay

SVF cells from AT or muscle were plated at 400 cells/cm² in 5 mL flasks with αMEM + 10%NCS + 1% ASP at 37 °C with 5% CO_2_. Medium was totally removed after 24 hours, and then partially changed every 2 days for 11 days. Staining of the CFU-F was performed with RAL stainer MCDh Kit (RAL Diagnostics) following manufacturer’s instructions.

### Adipogenesis assay

SVF cells from AT or muscle were plated at a density of 60 000 cells/cm² in αMEM + 10% NCS at 37 °C with 5% CO2. After 24 hours, medium was removed to eliminate the non-adherent cells, and replaced by adipogenic medium (αMEM, 2% NCS, Dexamethazone 33 nM, Insulin 5 mg/mL, Rosiglitazone 1 µM, T3 10 µM, Transferrin 10 µg/mL). After 4 days of incubation, medium was removed and cells were either fixed for further red oil staining or frozen at −20 °C for RNAs extraction.

### Cell migration assay

ASCs migration assay was performed using the IncuCyte® S3 Live-Cell Analysis System (v20192.3.7219.27517-1, 2019B Rev2 GUI, Essenbioscience). ASCs were isolated from mice ScAT or human samples as described above and were plated at a density of 2000 cell per well in a 96-well plate including a reservoir and an optically clear membrane insert assembly with laser-etched 8 µm pore. For mice migration assay, the reservoirs were loaded with 200 µl of plasma (50%) obtained from animals injured or not. For human migration assay, the reservoirs were loaded with 200 µl of human serum at 25% from healthy volunteers before or 1 hour after exercise. Cell migration across the pores was automatically quantified for over 30 h or 40 h for human and mouse ASCs respectively. Migration was analyzed via the Incucyte S3 software (Essenbioscience) and area under curves were calculated and compared using GraphPad Prism software v9 (GraphPad Software).

### Platelet/ASCs interaction assay

Freshly isolated ASCs were plated (80,000 cells/cm2) in αMEM + 10% NCS with 5% CO2. At ASCs confluence, 100.000 freshly harvested platelets stained with PKH26 (see above) and originating from ctrl or injured animals were added to ASCs for 1 hour (37 °C, 5%CO2). Then, medium was removed, cells were rinsed with phosphate-buffered saline (PBS) and fixed with 3,7% PFA. Nuclei were stained with DAPI. Acquisition and analysis of data were performed with Operetta® system type HH12 (Perkin Elmer). In order to evaluate the role of podoplanin on this cell interaction, anti-Podoplanin blocking antibody (Abcam, ab11936, 10 µg/mL) was added to the culture medium on ASCs before platelet addition.

### Immunohistochemistry

#### FAP/ASC immunohistology

Paraformaldehyde (PFA) (4%) fixed muscles and ScAT were embedded in agarose 2 to 3% for 1 hour and sliced with Vibroslicer® 5100 mz (Campden instruments) (300 µm thick). Samples were permeabilized with Triton X100 (0,2% in PBS) before non-specific antigen saturation with BSA 3% and Goat serum in PBS. Tissue sections were incubated with Goat anti-mouse CD140α (R&D AF1063, 1:100), rabbit anti-mouse CD45 (orb 10328 or sc-53665, 1:100), rat anti-mouse CD31 (BD 550274, 1:100), rat anti-mouse Sca-1 (BD 557403, 1:250), hamster anti-mouse podoplanin (Abcam 11936, 1:250), anti- GFP primary antibodies (Abcam ab1218 1:200) for 24 hours at 4 °C under constant agitation. Several washing with PBS were performed, and then sections were incubated with secondary antibodies (Donkey anti-goat 488/647, donkey anti-rabbit 594, goat anti-rabbit 647, donkey anti-rat 594, chicken anti-rat 647, goat anti-hamster 488/568, all from Invitrogen by Thermofisher Scientific, respective references A11058, A21447, A21207, A21245, A21209, A21472, A21110, A21112 and used at a dilution of at 1:250) for 8 hours at RT. After several washes, nucleus were stained with DAPI, and images were obtained using ZEISS LSM780 Confocal microscope (Zen Blue v2.3-2) and analyzed with Zen Blue v2.3-2, Fiji v2.1.0-1 and IMARIS® v 8 and 9.9.1 (Bitplane) software.

#### Regeneration evaluation

Mouse muscle samples were cryopreserved in OCT frozen in liquid nitrogen-cooled isopentane. Samples were then sectioned at 10 μm on a cryostat and post fixed with 4% PFA for 10 min at RT. After antigen retrieval step, slides were incubated with PBS containing Triton 0.5% (10 min, RT) and further permeabilized (TBS, glycine 100 mM, 0.1% Triton X100, 10 min, RT). They were blocked with 2% BSA (1 h, RT), TBS rinced, and stained overnight with WGA (Thermofisher, W11261, 1:500) (4 °C, moist chamber). After washing with TBS, sections were soaked for 10 min in DAPI solution and washed once with TBST and TBS before mounting with antifading mounting medium. Images were obtained with ZEISS LSM780 Confocal microscope and analyzed with, Fiji v2.1.0-1 and IMARIS® v8 and v9.9.1 (Bitplane) software. The size and distribution of myofibers with central nuclei was calculated from WGA–DAPI staining on all fibers of the section and area determination were performed across the entire sections using an automated image processing algorithm (Fiji v2.1.0-1).

#### IMCT quantification

Mouse muscles samples were carefully dissected at 14 dpi and fixed 24 h in PFA (4%). Muscles were then embedded in agarose 3% for 1 hour and sliced longitudinaly with Vibroslicer® 5100 mz (Campden instruments) (300 µm thick). Second Harmonic generation (SHG) of collagen fibers imaging was performed on muscle sections with a laser scanning microscope (LSM880; Carl Zeiss GmbH, Jena, Germany) combined with a pulsing titanium sapphire laser (Coherent, vissionII). xyz stacks images were acquired with a ×2.5 objective (N.A0.12, Carl Zeiss, GmbH) with mosaic tile mode to acquire the entire muscle section. The SHG signal of collagen fibers, and auto-fluorescence signals were detected at 830 nm excitation wavelength and respectively detected in a range of 408–420 and 446–695 nm. Muscle IMCT deposition was scored by determining the relative area of pixels exhibiting a significant SHG signal of the entire muscle section with a semi-segmentation process using the Fiji distribution (http://pacific.mpi-cbg.de/wiki/index.php/Fiji) of ImageJ (ImageJ-win64, Fiji v 2.1.0-1). 5–9 muscle sections were analyzed per animal in order to recapitulate the IMCT deposition of the entire quadriceps muscle.

### RNAs and genomic DNA extraction, and RT-qPCR

Extraction was performed on frozen cells or whole tissues using RNA Extractions Mini kit (QiaGEN) following manufacturer’s instructions. Briefly, samples were unfrozen in RLT and lysed with tissue lyser® (QIAGEN). Samples were passed through columns with washing steps to purify RNAs. Elution was performed with RNAse free water, and RNAs concentration was evaluated with Nanodrop® 2000c (Thermo Scientific).

Genomic DNA was obtained with QiaAmp DNA Mini Kit (QiaGEN) following manufacturer’s instructions. Cells were lysed and passed through column to bind DNA, and after two washing steps genomic material was eluted in Elution Buffer. Genomic DNA concentration was measured using n Nanodrop® 2000c; and then stored at −20 °C.

For qPCR, RNAs were reverse transcripted using High capacity reverse transcriptase (Invitrogen) and diluted at 50 ng/µL in RNAse-free water. Then, qPCR was performed using Fast SYBR® Green Mix (Applied Biosystems) on 96/384 wells plate, results were acquired with StepOne v2.3-3 and Viia7 devices (Life Technologies) and data were analyzed with Real-Time qPCR Studio (Life Technologies) using the 2−ΔΔCt method compared to Ctrl condition. All primer sequences are provided in a separate excel file called Supplementary Data 2.

### RNAseq

Total RNA was extracted using Zymo microprep kit (Ozyme) according to the manufacturer’s instructions. RNAseq (paired-end, 125 bp) was carried out by BGI Genomic Services using triplicates for each condition. Raw data were processed as follows. Sequencing reads were first filtered, using fastq_illumina_filter v 0.1, and quality control of filtered reads was performed using FastQC v 0.11.9. Filtered reads were then aligned onto the mm10 mouse genome using HISAT2 v 2.2.1 and counts per gene were quantified using featureCounts from Subread package v 2.0.1 on the mm10 transcripts annotation obtained from UCSC genome browser. Differential expression and statistical analyses were performed using DESeq2 v 1.34.0. To compare gene expressions, we used the following cutoffs: LOG2FC > 0.58 and *p* value <0.05. To generate heatmap, we selected the 3000 most variable genes using idep version 0.96. RNAseq data have been deposited under the accession number GSE214883.

### Single-cell RNAseq analysis

Data from Oprescu et al.^17^ and Malecova et al.^15^ were obtained and analyzed using Seurat package v 4.1.1.

### Human clinical study

Young active men (age 23.3 ± 1.7 years; BMI 23.3 ± 0.4 kg m^−2^; VO_2_ max 47.5 ± 1.6 ml kg^−1^ min^−1^) were recruited to take part in this study. The protocol was approved by the Dublin City University Ethics Committee and conducted in accordance with the criteria set by the Declaration of Helsinki; all subjects gave written informed consent. Participants were instructed to refrain from exercise and to replicate food intake the day before each trial. In the morning, following an overnight fast, participants lay on a bed for 1-hr after arriving at the lab. A blood sample was taken and they then exercised on a bicycle ergometer at 60% VO_2_ peak for 1-hr. VO2 peak was determined on a cycle ergometer starting at 70 watts and increasing in 30-watt increments every 3 minutes until exhaustion. A blood sample was taken at the end of the exercise. The intensity was determined using the results of an incremental exercise test to exhaustion. GDF-15 protein levels in blood samples were determined by ELISA (R&D Systems).

### Human AT samples

AT was obtained from patients who provided prior written informed consent according to the ethics committees of Toulouse Hospitals. AT was harvested during plastic surgery (abdominoplasty) from three adult patients (female, age 47.6 ± 3.7 years, BMI 26.7 ± 0.9 kg m^−2^) at Rangueil Hospital (part of CHU of Toulouse).

### Human AT-SVF isolation

Human AT was digested for 45 minutes in α-MEM (GIBCO) containing collagenase (NB4, Coger, 0.4 IU/mL), Dispase II (Roche, 1.6 UI/mL, Basel), in a water-shaking bath at 37 °C. After digestion, an equal volume of α-MEM was added to stop enzymatic digestion. The cells were passed through a 100-μm filter (Steriflip, Millipore, Billerica, MA) and then centrifuged. The pellet was resuspended in α-MEM containing 2% PLP and the total number of cells in the SVF was counted.

### Statistical analysis

Data are expressed as the mean ± s.e.m. Statistical analyses were performed using Student’s *t* test (two-sided paired or unpaired) or one-way ANOVA followed by the post hoc Dunnett’s and Tukey’s test with GraphPad Prism software v9 (GraphPad Software). *P* values less than *p < 0.05; **p < 0.01 and ***p < 0.001 were statistically significant.

### Reporting summary

Further information on research design is available in the Nature Portfolio Reporting Summary linked to this article.

## Supplementary information

Supplementary Information

Description of Additional Supplementary Files

Supplementary Data 1

Supplementary Data 2

Reporting Summary

## Data Availability

Data underlying Fig. 2A–E, H; 3A–C, F–H; 4A–C; 5A, C, E–G; 6B, C, E–H; 7B, C E–H, I are provided as Source Data files. The remaining data and Supplementary Figures are available within the article or from the authors upon reasonable request. Data from the human cohort of exercised patients in this study are not publicly available but can be requested as above. The RNAseq data of primary ASCs and FAPs generated in this study have been deposited in the NCBI’s GEO under accession code GSE214883. The single-cell RNAseq data from Oprescu et al.^17^ and Malecova et al.^15^ reanalyzed to generate Fig. 1E are accessible under accession codes GSE138826 and GSE100474, respectively. Confocal and Two-photon imaging datasets, which are several gigabytes, will be promptly made available upon request but are not immediately available for download due to file size. Source data are provided with this paper.

## Source data

Source Data
