# Supplementary material for: Adipose tissue is a source of regenerative cells that augment the repair of skeletal muscle after injury
Source: Nat Commun. 2023 Jan 5;14:80. doi: 10.1038/s41467-022-35524-7 (PMC9816314; doi:10.1038/s41467-022-35524-7)
Supplement: Supplementary file 1 — Supplementary Information [file 41467_2022_35524_MOESM1_ESM.pdf]

Adipose tissue is a source of regenerative cells that augment the repair of skeletal muscle after injury

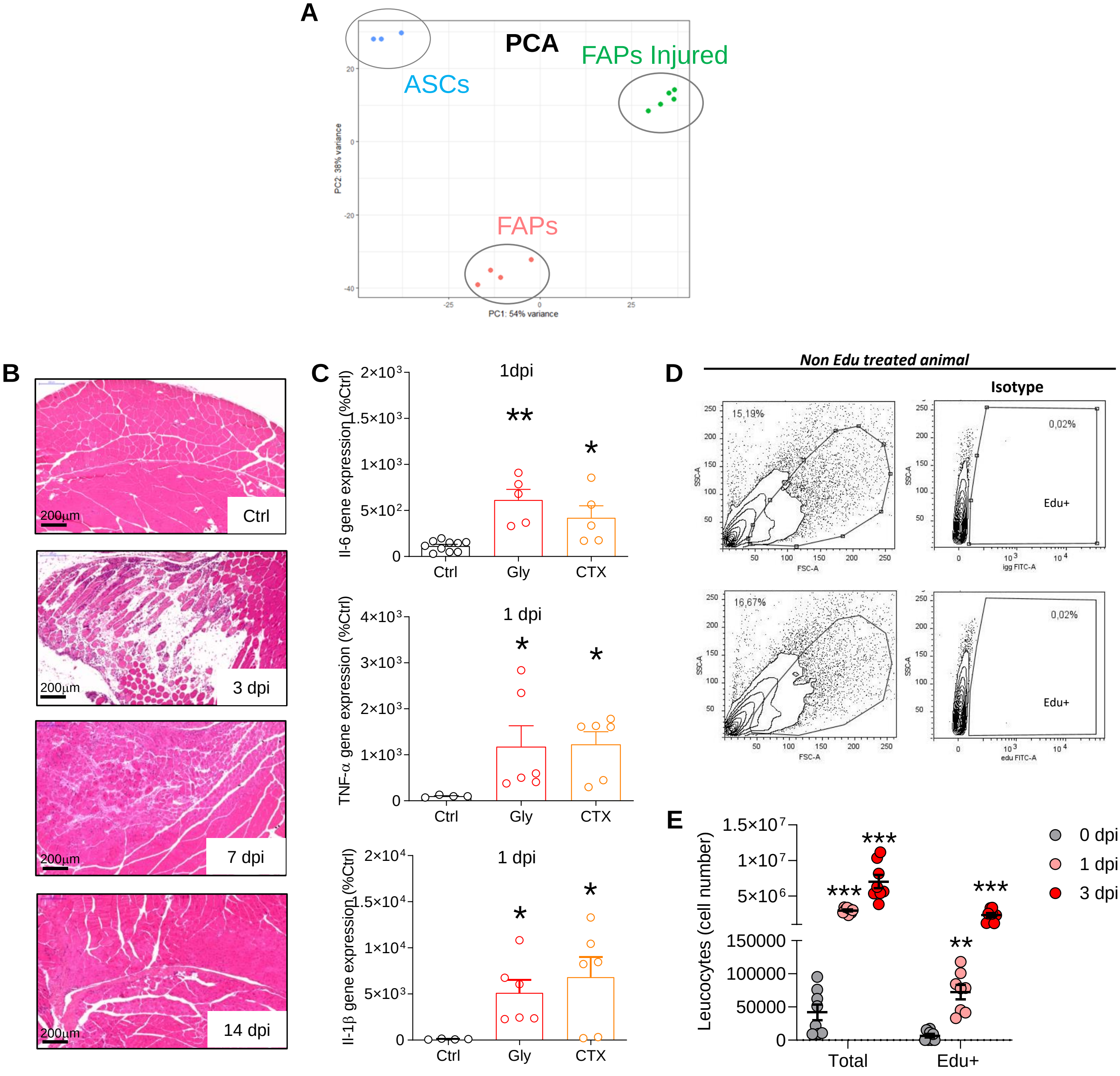

Figure S1 : Validation of mouse models of muscle injury

(A) Principal component analysis (PCA) of RNAseq expression values of FAPs and ASCs isolated from injured (1 dpi) and control animals. (B) Representative images of Hemalun-Eosine stained muscles from control uninjured mice (0 dpi) and during muscle regeneration (Gly, 3 to 14 dpi). Regenerating fibers are detected with the presence of centronuclei. Bar scale 200µm. (C) Expression of proinflammatory cytokines at 1 dpi in injured muscles (Gly, CTX) compared to uninjured muscle (Ctrl). n= 4-10 (Ctrl), 5-6 (Gly or CTX) animals over 4 independent experiments. (D) Representative image of positive and negative gates which were set by analysing isotype or unstained control sample in each analysis. (E) Comparison of *in vivo* EdU incorporation in FAPs by flow cytometry after Gly damage at 1 dpi. n=8 animals at all time points over 3 independent experiments. Results are expressed as mean ± SEM; *p<0.05, **p<0.01, ***p<0.001 vs Ctrl.

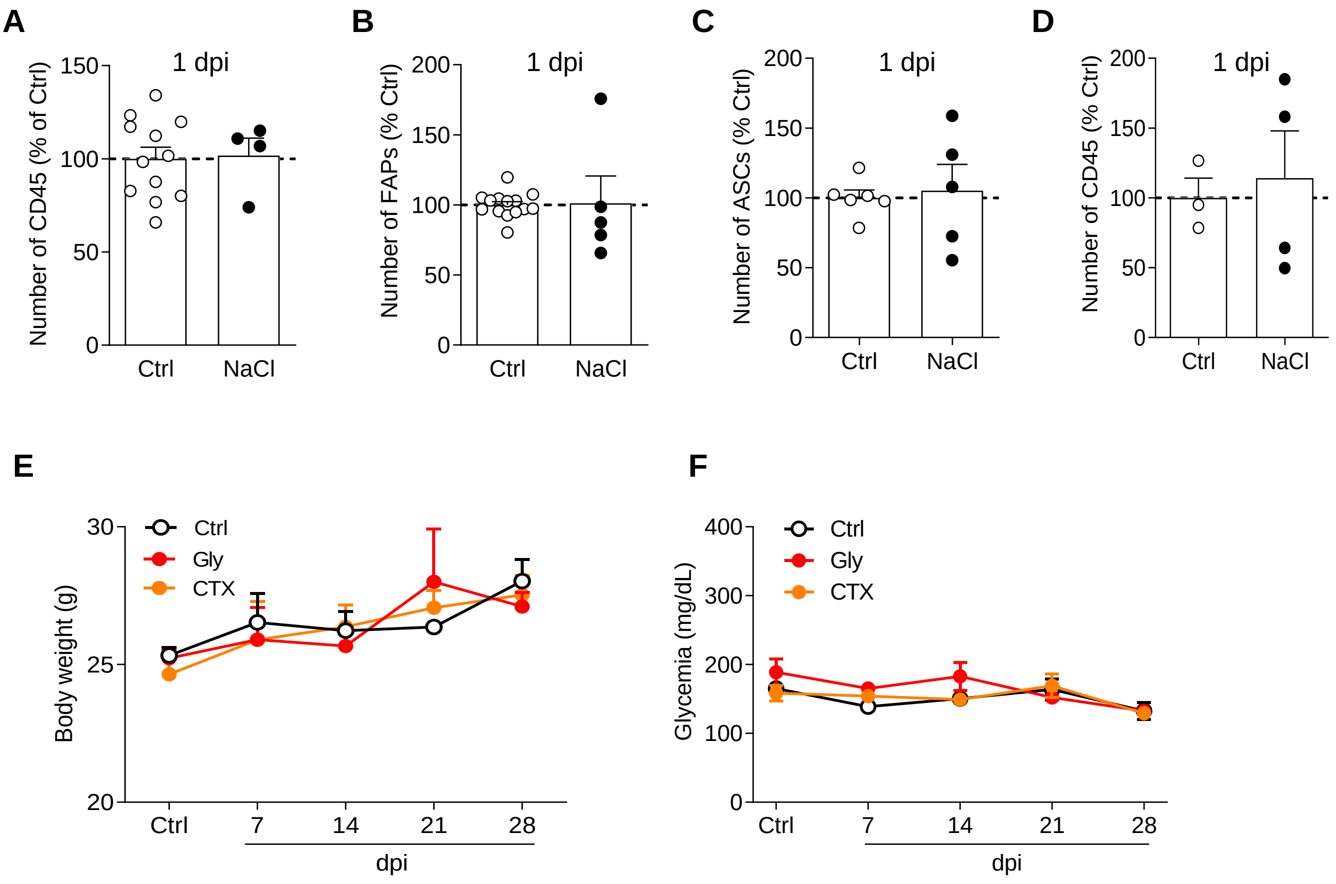

Figure S2 : Both FAP increase and ASCs decrease are injury-dependent effects and do not modify body weight and glycemia of the animals.
At 1 dpi with NaCl 0,9% injected in the quadriceps muscle, $CD45^+$ cells (A) and FAPs (B) were quantified in the muscle by flow cytometry analyses. For A, n= 12(Ctrl), 4 (NaCl) animals of 2 independent experiments. For B, n= 14 (Ctrl), 5 (NaCl) animals of 2 independent experiments. ASCs (C) or $CD45^+$ (D) cells from the ScAT were quantified by flow cytometry analyses at 1dpi with NaCl 0.9% injection in the quadriceps muscle. For C, n= 6 (Ctrl), 5 (NaCl) animals of 2 independent experiments. For D, n= 3 (Ctrl), 4 (NaCl) animals of 2 independent experiments. Time course of body weight (E) and glycemia (F) in uninjured (Ctrl) or muscle injured (Gly or CTX). For E and F, n=3 animals for all groups at each time point. Results are expressed as mean ± SEM. Statistical significance is set at $p \leq 0.05$.

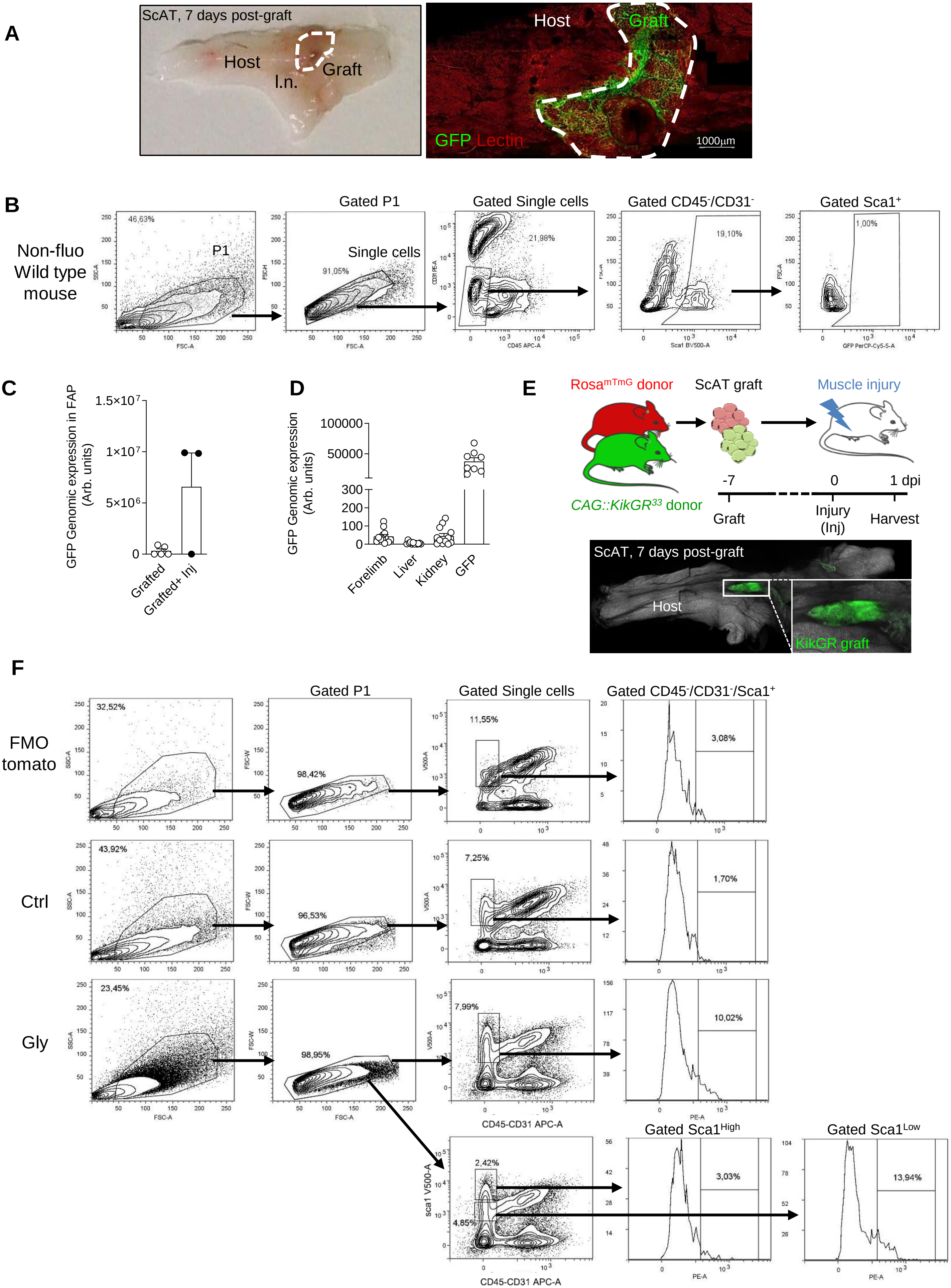
A
ScAT, 7 days post-graft
Host
Graft
l.n.
Host
Graft
GFP Lectin
1000µm
B
Non-fluo
Wild type
mouse
46,63%
P1
FSC-A
SSC-A
Gated P1
Single cells
91,05%
FSC-H
FSC-A
Gated Single cells
21,98%
CD31 PE-A
CD45 APC-A
Gated CD45-/CD31-
19,10%
FSC-A
Sca1 BV500-A
Gated Sca1+
1,00%
FSC-A
GFP PerCP-Cy5-5-A
C
GFP Genomic expression in FAP
(Arb. units)
1.5×10⁷
1×10⁷
5×10⁶
0
Grafted
Grafted+ Inj
D
GFP Genomic expression
(Arb. units)
100000
50000
300
200
100
0
Forelimb
Liver
Kidney
GFP
E
RosamTmG donor
ScAT graft
Muscle injury
CAG::KikGR33 donor
-7
0
1 dpi
Graft
Injury
(Inj)
Harvest
ScAT, 7 days post-graft
Host
KikGR graft
F
FMO
tomato
Ctrl
Gly
Gated P1
Gated Single cells
Gated CD45-/CD31-/Sca1+
32,52%
98,42%
11,55%
3,08%
43,92%
96,53%
7,25%
1,70%
23,45%
98,95%
7,99%
10,02%
SSC-A
FSC-W
V500-A
FSC-A
CD45-CD31 APC-A
PE-A
Gated Sca1High
Gated Sca1Low
2,42%
4,85%
3,03%
13,94%
Sca1 V500-A

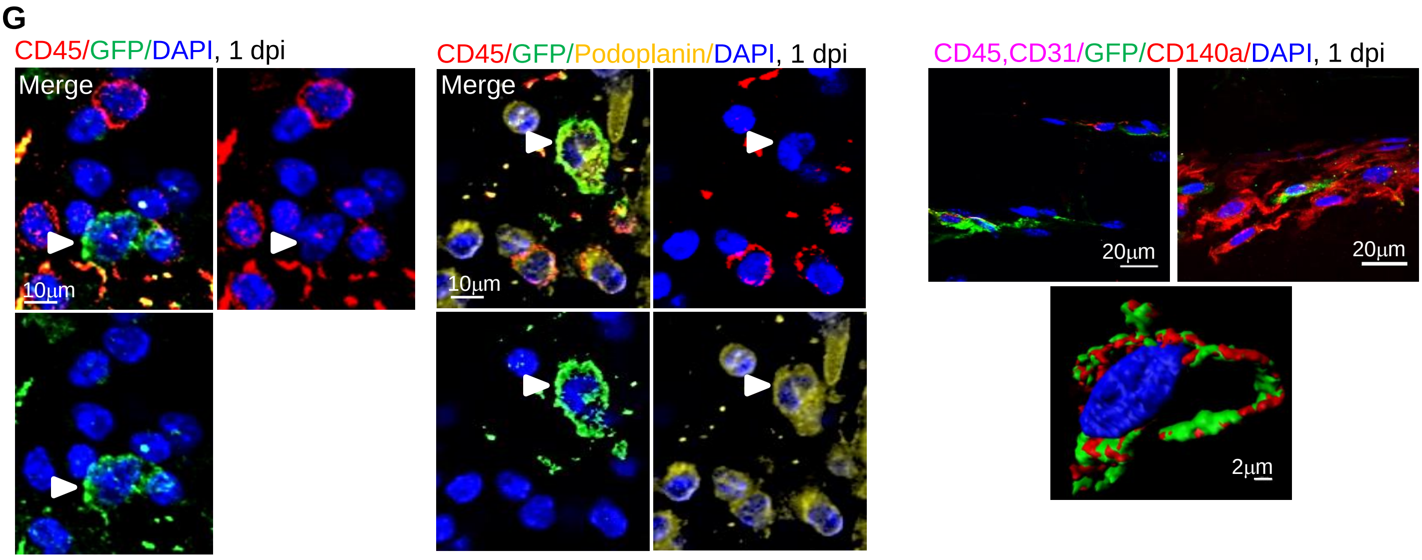

Figure S3 : Validation of the fat grafting model.

(A) Representative image of grafted ScAT (delimited by the white dashed line) inserted in the host adipose tissue (left panel). Fluorescent image was performed to visualize functional revascularization with lectin staining (red) injected *in vivo* 7 days after the grafting (right panel). Bar scale : 1 mm. (B) Gating strategy of muscle-derived SVFs from CD34-GFP grafted animals which were glycerol injured (Inj-Gly) or not (Ctrl), here is presented a non-fluorescent animal as an internal cytometry control. (C- D) GFP genomic expression analysis of $CD34^{+}$-GFP cells infiltrated in organs of grafted mice at 1 dpi. For C, n= 5 (grafted) and 3 (grafted+Gly) animals over 2 independent experiments. For D, n=12 (Forelimb, liver and kidney) and 8 (GFP) animals over 4 independent experiments. (E) Model of ScAT grafting from $Rpsa26^{mT/mG}$ (red) or CAG:KikGR (green) mouse into WT C57Bl/6 mice, the figure was partly generated using Servier Medical Art, provided by Servier, licensed under a Creative Commons Attribution 3.0 unported license. (F) Flow cytometry analysis of the SVF from injured muscle of the mT/mG grafted mice (1 dpi). (G) Immunohistological confocal images of injured quadriceps in CD34-GFP grafted mice at 1 dpi *in situ* (arrowheads point $CD34\text{-}GFP^{+}/Podoplanin^{+}/CD45^{-}$ or $CD34\text{-}GFP^{+}/CD140a^{+}/CD31^{-}/CD45^{-}$ cells). Bar scales, 10 um (left and middle panels), 20 and 2 um (right panel). Results are expressed as mean ± SEM. Statistical significance is set at $p \leq 0.05$.

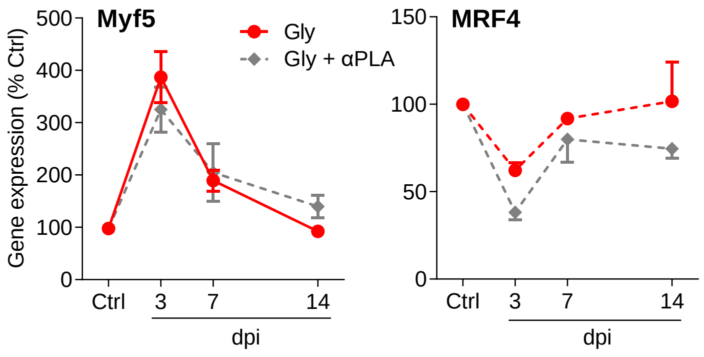

Figure S4 : Effect of platelet depletion on gene expression of early markers of muscle regeneration
(A) Time course expression of myogenic genes in quadriceps muscle from injured animals in the presence (Gly) or absence (Gly + αPL) of platelets. n= 8-14 (Ctrl), 3-9 (Gly), 4-6 (Gly+αPLA) animals over 4 independent experiments. Results are expressed as mean ± SEM. Statistical significance is set at $p \leq 0.05$.

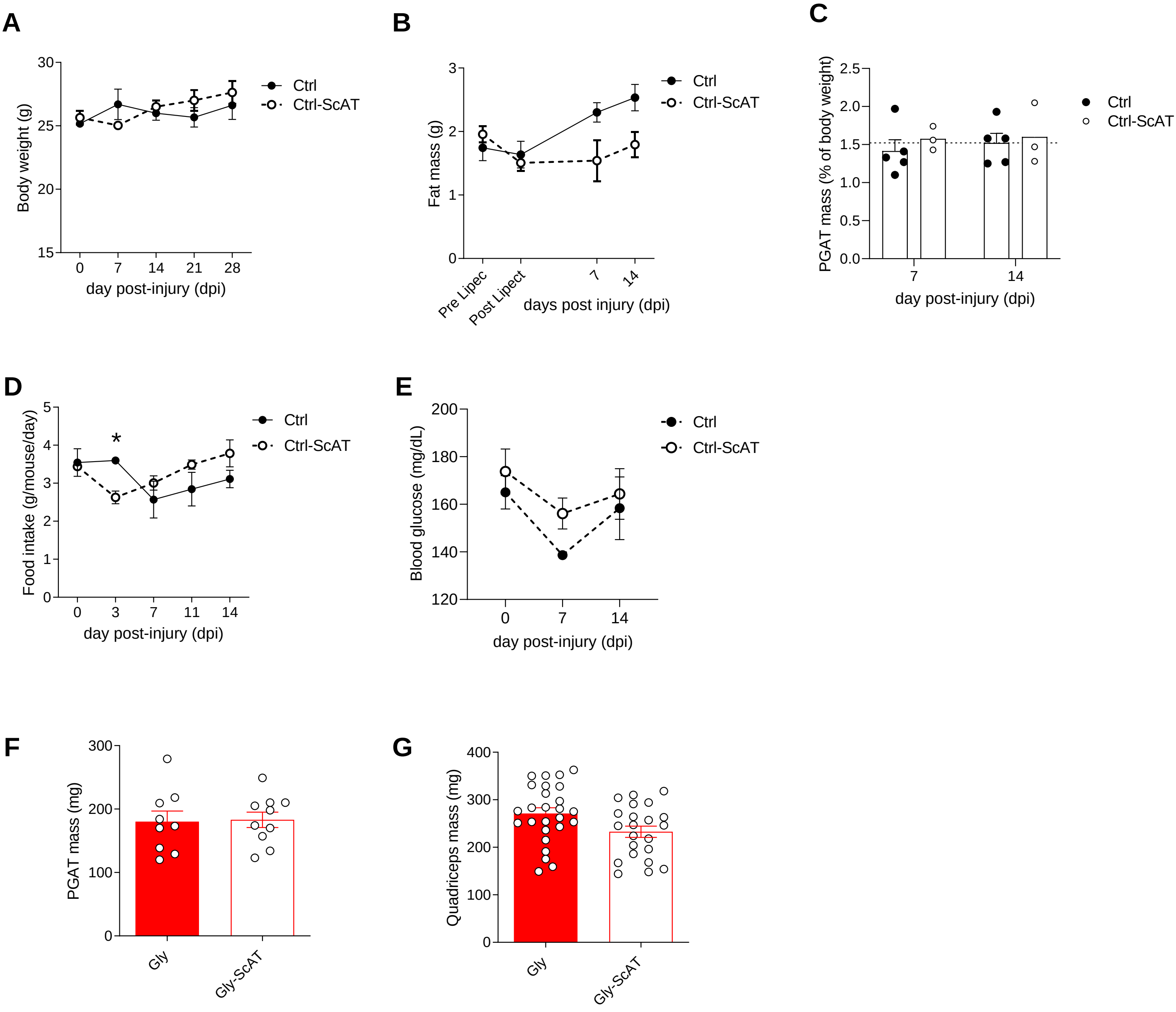

Figure S5: Time courses of body weight (A), fat mass (B), Perigonadal adipose tissue (PGAT) mass (C) food intake (D) and blood glucose (E) in sham control and lipectomied animals up to 28 days post-surgery.

For A-E, n= 3-5 (Ctrl), 3-9 (Ctrl-ScAT) animals over 3 independent experiments. PGAT (F) and Quadriceps muscle (G) mass in sham and lipectomied animals 1 day post muscle injury with glycerol. For F-G n=9-16 (Ctrl), 10-22 (Ctrl-ScAT) animals over 5 independent experiments.
