# Supplementary material for: Adipose tissue is a source of regenerative cells that augment the repair of skeletal muscle after injury
Source: Nat Commun. 2023 Jan 5;14:80. doi: 10.1038/s41467-022-35524-7 (PMC9816314; doi:10.1038/s41467-022-35524-7)
Supplement: Supplementary file 2 — Description of Additional Supplementary Files [file 41467_2022_35524_MOESM2_ESM.pdf]

Description of Additional Supplementary Files

File Name: Supplementary Data 1
Description: List of the GO-biological process enriched in ScAT-ASCs compared to PGAT-ASCs.

File Name : Supplementary Data 2
Description : Sequences of the primers used in the study.
