# Supplementary material for: Adipose tissue is a source of regenerative cells that augment the repair of skeletal muscle after injury
Source: Nat Commun. 2023 Jan 5;14:80. doi: 10.1038/s41467-022-35524-7 (PMC9816314; doi:10.1038/s41467-022-35524-7)
Supplement: Supplementary file 5 — Reporting Summary [file 41467_2022_35524_MOESM5_ESM.pdf]

nature portfolio

Corresponding author(s): Coralie SENGENES

Last updated by author(s): Nov 10, 2022

Reporting Summary

Nature Portfolio wishes to improve the reproducibility of the work that we publish. This form provides structure for consistency and transparency in reporting. For further information on Nature Portfolio policies, see our Editorial Policies and the Editorial Policy Checklist.

Statistics

For all statistical analyses, confirm that the following items are present in the figure legend, table legend, main text, or Methods section.

n/a	Confirmed	
☐	☒	The exact sample size (*n*) for each experimental group/condition, given as a discrete number and unit of measurement
☐	☒	A statement on whether measurements were taken from distinct samples or whether the same sample was measured repeatedly
☐	☒	The statistical test(s) used AND whether they are one- or two-sided<br>*Only common tests should be described solely by name; describe more complex techniques in the Methods section.*
☒	☐	A description of all covariates tested
☐	☒	A description of any assumptions or corrections, such as tests of normality and adjustment for multiple comparisons
☐	☒	A full description of the statistical parameters including central tendency (e.g. means) or other basic estimates (e.g. regression coefficient) AND variation (e.g. standard deviation) or associated estimates of uncertainty (e.g. confidence intervals)
☐	☒	For null hypothesis testing, the test statistic (e.g. *F*, *t*, *r*) with confidence intervals, effect sizes, degrees of freedom and *P* value noted<br>*Give P values as exact values whenever suitable.*
☒	☐	For Bayesian analysis, information on the choice of priors and Markov chain Monte Carlo settings
☒	☐	For hierarchical and complex designs, identification of the appropriate level for tests and full reporting of outcomes
☒	☐	Estimates of effect sizes (e.g. Cohen's *d*, Pearson's *r*), indicating how they were calculated

Our web collection on statistics for biologists contains articles on many of the points above.

Software and code

Policy information about availability of computer code

Data collection

- FACSDiva version 9.0.1 (CST Version 3.5, PLA Version 2.0): flow cytometry acquisition software
- Zen Blue version 2.3-2: Imaging acquisition software.
- Zen Black version 2.3SP1:Imaging acquisition software.
- StepOne version 2.3-3: collect data of real-time quantitative PCR
- Viia7 version: collect data of real-time quantitative PCR
- IncuCyte 20192.3.7219.27517-1, 2019B Rev2 GUI (Essen BioScience): Flexible, real-time imaging and analysis of live-cell biology, used to quantify cell migration activity.
- Operetta CLS type HH12 – High Content Imaging System PERKIN ELMER: Wide field or confocal XYZT acquisition, multi-colour fluorescence or transmitted light, imaging acquisition of cultured cells and muscle stained slices.

Data analysis

- FACSDiva version 9.0.1 : flow cytometry data analysis
- FlowLogic version 8.1-2: flow cytometry data analysis
- FlowJo version 10.8: flow cytometry data analysis
- Zen Black version 2.3SP1:Imaging acquisition software
- Zen Blue version 2.3-2: Imaging analysis software
- Fiji version 2.1.0-1: Imaging analysis software
- IMARIS® version 8 and 9.9.1-2 (Bitplane): Imaging analysis software
- StepOne version 2.3-3: RTqPCR data analysis
- Seurat package version 4.1.1: Single cell RNAseq analysis
- IncuCyte 20192.3.7219.27517-1, 2019B Rev2 GUI (Essen BioScience): cell migration analysis
- GraphPad Prism version 9: Stastistics and graphs
- Harmony software version 4.5 (Perkin Elmer): Automated image processing algorithm for muscle fiber size/quantification
- Desq2- bioconductor.org: ersion 1.34.0 : differential gene expression analysis
- FeatureCounts (Package Subread version 2.0.1): sequencing reads assignment

- Fastq_illumina_filter version 0.1: filtration of sequencing reads
- FastQC version 0.11.9 : quality control of sequencing reads
- HISAT2 version 2.2.1: alignment of sequenced reads with mouse genome
- idep version 0.96: heatmap generation

For manuscripts utilizing custom algorithms or software that are central to the research but not yet described in published literature, software must be made available to editors and reviewers. We strongly encourage code deposition in a community repository (e.g. GitHub). See the Nature Portfolio guidelines for submitting code & software for further information.

Data

Policy information about availability of data

All manuscripts must include a data availability statement. This statement should provide the following information, where applicable:

- Accession codes, unique identifiers, or web links for publicly available datasets
- A description of any restrictions on data availability
- For clinical datasets or third party data, please ensure that the statement adheres to our policy

The data that support the plots within this paper and other findings of this study are available from the corresponding author upon reasonable request.

Field-specific reporting

Please select the one below that is the best fit for your research. If you are not sure, read the appropriate sections before making your selection.

☒ Life sciences ☐ Behavioural & social sciences ☐ Ecological, evolutionary & environmental sciences

For a reference copy of the document with all sections, see nature.com/documents/nr-reporting-summary-flat.pdf

Life sciences study design

All studies must disclose on these points even when the disclosure is negative.

Sample size	Animal studies : No sample size calculation was performed. For phenotypic analyses, we followed guidelines from Institut Clinique de la Souris (http://www.ics-mci.fr), Mouse Phenome Data Base (http://phenome.jax.org/) and International Mouse Phenotyping Consortium (http://www.mousephenotype.org/).<br>RNAseq experiment: for each cell type (control and injured ASCs and FAPs) triplicates were made from a pool of cells isolated from 3 different animals in order to represent interindividual variability. No sample size calculation was performed but triplicate is a standard size commonly accepted for this type of experiment. Cost and number of samples per sequencing lanes were limiting factors.<br>Mouse In vitro experiments: No sample size calculation was performed however we knew from our previous published work that the chosen size was sufficient for the observation.<br>Human in vitro experiments: No sample size calculation was performed. We used all the available 6 individuals and from their tissues we performed several primary cell preparations.
Data exclusions	Data exclusion had to be done for very few figures. When done, identified outlier values have been verified with the "Outlier calculator" tool from GraphPad Prism v9. For exemple, we had to exclude some data from animals where muscle lesion was not done properly (we could check that each time thanks to the measurement of the CD45 positive cell population in flow cytometry).
Replication	Data reported in the manuscript are from biological replicates.<br>Animal studies: Animals from several litters were used in each experiment to avoid litter-to-litter variation. For all experiments studying the effects of muscle injury at 1 dpi, each experiments included at least 3 animals per group, and experiments were replicated at least on 3 independent days. For experiments using anti-platelets, lipectomy and cell reinjection, due to ethical concerns we have made the experiments with 2 batch of animals, replicated twice only. All attempts were successful (unless the muscle lesion was fully effective, see data exclusion above).<br>Scat-Graft experiments initially performed with a CD34GFP mouse donor were replicated with others fluorescent mouse models (mT/mG and KikGR) and replicated more than 3 times, on differents days.<br>Cell studies: In studies on murine ASCs, reproducibility of measured effects is high when replicates are performed on different wells from the same primary cell culture preparation. Results were also replicated on different primary cell culture preparation on different days. All attempts were successful. Concerning studies with human ASCs (also primary cell culture), results were replicated with several donor tested on the same serum to measure their migration activity.
Randomization	Animal studies : Randomization of the mice allocation into experimental groups (muscle Glycerol or CTX lesion/ unijured control).<br>Cell studies: No randomization was performed however 3 differents operators obtained similar results. We thus considered our results reliable.<br>Human studies: No randomization in group comparisons, all subjects were measured before and after exercice. Randomization was not relevant here.
Blinding	Animal studies: Experiments were not blinded. Most of the time the same operators performed muscle lesion, animal tissues dissection and primary cell preparation. It was thus impossible to perform blinding. Whenever possible we performed blinding. Thus, the nature of the groups was blinded to investigators performing muscle IHC analyses and muscle fibres size quantification.<br>Cell studies : Experiments were not blinded. Blinding would have required more independant operators to perform the experiment and most of the time, the operator is able to recognize the sample group due to tissue color/ cell density etc making blinding less effective anyway.

Human studies: Nature of the groups was blinded to the investigator performing cell migration assay.
For animal studies, most of the time the same operators performed muscle lesion, animal tissues dissection and primary cell preparation. It was thus impossible to perform blinding.

Behavioural & social sciences study design

All studies must disclose on these points even when the disclosure is negative.

Study description	*Briefly describe the study type including whether data are quantitative, qualitative, or mixed-methods (e.g. qualitative cross-sectional, quantitative experimental, mixed-methods case study).*
Research sample	*State the research sample (e.g. Harvard university undergraduates, villagers in rural India) and provide relevant demographic information (e.g. age, sex) and indicate whether the sample is representative. Provide a rationale for the study sample chosen. For studies involving existing datasets, please describe the dataset and source.*
Sampling strategy	*Describe the sampling procedure (e.g. random, snowball, stratified, convenience). Describe the statistical methods that were used to predetermine sample size OR if no sample-size calculation was performed, describe how sample sizes were chosen and provide a rationale for why these sample sizes are sufficient. For qualitative data, please indicate whether data saturation was considered, and what criteria were used to decide that no further sampling was needed.*
Data collection	*Provide details about the data collection procedure, including the instruments or devices used to record the data (e.g. pen and paper, computer, eye tracker, video or audio equipment) whether anyone was present besides the participant(s) and the researcher, and whether the researcher was blind to experimental condition and/or the study hypothesis during data collection.*
Timing	*Indicate the start and stop dates of data collection. If there is a gap between collection periods, state the dates for each sample cohort.*
Data exclusions	*If no data were excluded from the analyses, state so OR if data were excluded, provide the exact number of exclusions and the rationale behind them, indicating whether exclusion criteria were pre-established.*
Non-participation	*State how many participants dropped out/declined participation and the reason(s) given OR provide response rate OR state that no participants dropped out/declined participation.*
Randomization	*If participants were not allocated into experimental groups, state so OR describe how participants were allocated to groups, and if allocation was not random, describe how covariates were controlled.*

Ecological, evolutionary & environmental sciences study design

All studies must disclose on these points even when the disclosure is negative.

Study description	*Briefly describe the study. For quantitative data include treatment factors and interactions, design structure (e.g. factorial, nested, hierarchical), nature and number of experimental units and replicates.*
Research sample	*Describe the research sample (e.g. a group of tagged Passer domesticus, all Stenocereus thurberi within Organ Pipe Cactus National Monument), and provide a rationale for the sample choice. When relevant, describe the organism taxa, source, sex, age range and any manipulations. State what population the sample is meant to represent when applicable. For studies involving existing datasets, describe the data and its source.*
Sampling strategy	*Note the sampling procedure. Describe the statistical methods that were used to predetermine sample size OR if no sample-size calculation was performed, describe how sample sizes were chosen and provide a rationale for why these sample sizes are sufficient.*
Data collection	*Describe the data collection procedure, including who recorded the data and how.*
Timing and spatial scale	*Indicate the start and stop dates of data collection, noting the frequency and periodicity of sampling and providing a rationale for these choices. If there is a gap between collection periods, state the dates for each sample cohort. Specify the spatial scale from which the data are taken*
Data exclusions	*If no data were excluded from the analyses, state so OR if data were excluded, describe the exclusions and the rationale behind them, indicating whether exclusion criteria were pre-established.*
Reproducibility	*Describe the measures taken to verify the reproducibility of experimental findings. For each experiment, note whether any attempts to repeat the experiment failed OR state that all attempts to repeat the experiment were successful.*
Randomization	*Describe how samples/organisms/participants were allocated into groups. If allocation was not random, describe how covariates were controlled. If this is not relevant to your study, explain why.*
Blinding	*Describe the extent of blinding used during data acquisition and analysis. If blinding was not possible, describe why OR explain why blinding was not relevant to your study.*

Did the study involve field work? ☐ Yes ☐ No

Field work, collection and transport

Field conditions	*Describe the study conditions for field work, providing relevant parameters (e.g. temperature, rainfall).*
Location	*State the location of the sampling or experiment, providing relevant parameters (e.g. latitude and longitude, elevation, water depth).*
Access & import/export	*Describe the efforts you have made to access habitats and to collect and import/export your samples in a responsible manner and in compliance with local, national and international laws, noting any permits that were obtained (give the name of the issuing authority, the date of issue, and any identifying information).*
Disturbance	*Describe any disturbance caused by the study and how it was minimized.*

Reporting for specific materials, systems and methods

We require information from authors about some types of materials, experimental systems and methods used in many studies. Here, indicate whether each material, system or method listed is relevant to your study. If you are not sure if a list item applies to your research, read the appropriate section before selecting a response.

Materials & experimental systems

n/a	Involved in the study	
☐	☒	Antibodies
☒	☐	Eukaryotic cell lines
☒	☐	Palaeontology and archaeology
☐	☒	Animals and other organisms
☐	☒	Human research participants
☐	☒	Clinical data
☒	☐	Dual use research of concern

Methods

n/a	Involved in the study	
☒	☐	ChIP-seq
☐	☒	Flow cytometry
☒	☐	MRI-based neuroimaging

Antibodies

Antibodies used

Antibody / Supplier /Catalogue /Clone / Lot :
Primary Antibodies :
Rat monoclonal against CD45 / Santa cruz biotechnology / Cat# sc-53665 / 30-F11 / A#0814
Rabbit polyclonal against CD45/ Biorbyt / Cat#orb10328 / A0684
Rat monoclonal against CD31 / BD biosciences / Cat# BD550274 / MEC13.3 / 09827
Rat monoclonal against Sca1 / BD biosciences / Cat# 557403 / D7 / 3315918
Syrian Hamster monoclonal [RTD4E10] against podoplanin, gp36 / Abcam / Cat# Ab11936 / n/a / GR3172946-3
Goat polyclonal against CD140a / R&D Systems / Cat# AF1062 / n/a / HMQ0218081
Mouse monoclonal against GFP / Abcam / Cat# ab1218 /n/a /
Wheat Germ Agglutinin, Alexa 488 / Invitrogen / Cat#W11261 / 2411584

Secondary Antibodies: (all from Invitrogen by Thermofisher Scientific)
Donkey anti-Goat Alexa 594 / Molecular probes / Cat# A 11058 / 1736986
Donkey anti-Goat Alexa 647 / Molecular probes / Cat# A 21447/
Goat anti-hamster Alexa 488 / Molecular probes / Cat# A21110 / 2041067
Goat anti-hamster Alexa 568 / Molecular probes / Cat# A21112 / 1700388
Donkey anti-mouse Alexa 488 / Molecular probes / Cat# A 21202 / 1915874
Donkey anti-Rabbit Alexa 488 / Molecular probes / Cat# A 21206 / 2072687
Donkey anti-Rabbit Alexa 594 / Molecular probes / Cat# A 21207 / 2266563
Goat anti-Rabbit Alexa 647/ Molecular probes / Cat# A 21245/ 2018272
Goat anti-rat Alexa 488 / Molecular probes / Cat# A11006 / 1825822
Donkey anti-Rat Alexa 594 / Molecular probes / Cat# A 21209 /1807726
Chicken anti-rat Alexa 647 / Molecular probes / Cat# A21472 / 1746276

Antibodies for flow cytometry:
CD31-PE /BD Biosciences / #553373 / clone MEC13-3
CD45-PE /BD Biosciences / #553081/ clone 30F11
Podoplanin-APC / Biolegend / #127410 / clone 8.1.1
CD140a-APC / Biolegend / #135908 / clone APA5
CD31-FITC /BD Biosciences / #553372 / clone MEC13-3
CD45-FITC /BD Biosciences / #553080/ clone 30F11
CD34-FITC /BD Biosciences / #553733 / clone RAM34
Sca1-FITC /BD Biosciences / #553335/ clone E16-161.17
Sca1-BV500 /BD Biosciences / #561228/ clone D7
Rat Isotype PE IgG2a/BD Biosciences / #553930 / clone R35-95
Mouse Isotype APC / Biolegend / #402012 / clone SHG-1
Rat Isotype APC IgG2a /BD Biosciences / #553932/ clone R35-95
Rat Isotype FITC IgG2a /BD Biosciences / #554688 / clone R35-95

Rat Isotype BV500 IgG2a /BD Biosciences / #560786 / R35-95
CD45-PerCP-Vio700 / Miltenyi / 130-110-801 / clone REA737
CD31-PE/ Miltenyi / 130-111-540 / clone REA784
CD34-FITC / Miltenyi / 130-117-775 / clone REA383
Sca1-PE-Vio770 / Miltenyi / 130-106-258 / clone REA422
CD140a-APC-Vio770 / Miltenyi / 130-125-991 / clone REA637
CD90.2-VioBlue / Miltenyi / 130-102-345/ clone 30-H12
Isotype PerCP-Vio700 / Miltenyi / 130-113-453 / clone REA293
Isotype PE / Miltenyi / 130-113-450 / clone REA293
Isotype FITC / Miltenyi / 130-113-449 / clone REA293
Isotype PE-Vio770 / Miltenyi / 130-113-452 / clone REA293
Isotype APC-Vio770 / Miltenyi / 130-113-447 / clone REA293
Rat Isotype VioBlue IgG2b / Miltenyi / 130-102-661 / clone ES26-5E12.4

Antibodies for ASC/FAP sorting:
CD31-FITC / Miltenyi / 130-123-675/ clone 390
CD45-FITC / Miltenyi / 130-116-535 / clone 30F11
anti-FITC magnetic microbeads / Miltenyi / 130-048-701
anti-Sca-1 magnetic microbeads / Miltenyi / 130-106-641

Antibodies used to treat animals in vivo:
platelet depleting antibody: anti-GPIb /EMFRET/ #R300
Control rat IgG: mixture of non-immune rat IgG which display no cytotoxic effects on platelets in mice /EMFRET/ #C301
anti podoplanin / BioCell / #BE0236 / clone 8.1.1
Control polyclonal Syrian hamster IgG / BioCell / #BE0087

Validation

- Rat monoclonal against CD31 / BD biosciences / Cat# BD550274 / MEC13.3 : validation statement on manufacturer website https://www.bdbiosciences.com/en-us/products/reagents/flow-cytometry-reagents/research-reagents/single-color-antibodies-ruo/purified-rat-anti-mouse-cd31.550274, "tested on cardiac muscle, staining endothelial cells on small and large blood vessels". Validated in previous publication of the lab DOI: 10.1016/j.celrep.2019.03.038.
- Rat monoclonal against CD45 / Santa cruz biotechnology / Cat# sc-53665 / 30-F11: validation on manufacturer website https://www.scbt.com/fr/p/cd45-antibody-30-f11, "CD45 (30-F11) is recommended for detection of CD45 of mouse origin by immunohistochemistry (including paraffin-embedded sections)(starting dilution 1:50, dilution range 1:50-1:500)" , the manufacturer provides 16 publications using this Ab, including our previous work DOI: 10.1016/j.celrep.2019.03.038.
- Rat polyclonal against CD45 / Biorbyt / Cat#orb10328 / A0684: the manufacturer provides several images of IHC-P images of different mouse tissues (skin, lung, pancreas) and said that several dilutions have been tested (range 1:100-1:500).
- Rat monoclonal against Sca1 / BD biosciences / Cat# 557403 / D6 : this antibody has previously been validated in works of the laboratory, in murine muscle DOI: 10.1016/j.celrep.2019.03.038.
- Syrian Hamster monoclonal [RTD4E10] against podoplanin, gp36 / Abcam / Cat# Ab11936 / n/a : validation on manufacturer website https://www.abcam.com/podoplanin--gp36-antibody-rtd4e10-bsa-and-azide-free-ab11936.html. "The monoclonal antibody will detect podoplanin on the surface of mouse lymphatic endothelial cells and some epithelial cell types by immunostaining or immunohistochemistry". Ab promise guarantee covers the use of ab11936 in IHC-P tested applications. Ab has been validated in house on murine muscles.
- Goat polyclonal against CD140a / R&D Systems / Cat# AF1062 / n/a : manufacturer website provides validation on whole mouse embryo (thus including muscles) https://www.rndsystems.com/products/mouse-pdgf-ralpha-antibody_af1062. Ab has been validated in house on murine muscles.
- Mouse monoclonal against GFP / Abcam / Cat# ab1218 /9F9.F9: validation on manufacturer website https://www.abcam.com/gfp-antibody-9f9f9-ab1218.html. Ab promise guarantee covers the use of ab1218 in IHC tested applications. Ab is cited in 303 references including for IHC purposes. Ab has been validated in house on murine muscles.

All primary antibodies used in this article are either already used in routine and have been used in prior published works or tested on muscle tissue slices to determine optimal experimental conditions (dilution, Ab recovery etc...).
All secondary antibodies used in this article are already used in routine and have been used in prior published works of the laboratory. However, we always re-test their specificity by incubating them alone without primary Ab first.

Eukaryotic cell lines

Policy information about cell lines

Cell line source(s): *Only primary cell cultures have been used in this article. No cell line has been used*

Authentication: *No cell line has been used*

Mycoplasma contamination: *No cell line has been used*

Commonly misidentified lines (See ICLAC register): *No cell line has been used*

Palaeontology and Archaeology

Specimen provenance: *Provide provenance information for specimens and describe permits that were obtained for the work (including the name of the issuing authority, the date of issue, and any identifying information). Permits should encompass collection and, where applicable, export.*

Specimen deposition | *Indicate where the specimens have been deposited to permit free access by other researchers.*

Dating methods | *If new dates are provided, describe how they were obtained (e.g. collection, storage, sample pretreatment and measurement), where they were obtained (i.e. lab name), the calibration program and the protocol for quality assurance OR state that no new dates are provided.*

☐ Tick this box to confirm that the raw and calibrated dates are available in the paper or in Supplementary Information.

Ethics oversight | *Identify the organization(s) that approved or provided guidance on the study protocol, OR state that no ethical approval or guidance was required and explain why not.*

Note that full information on the approval of the study protocol must also be provided in the manuscript.

Animals and other organisms

Policy information about studies involving animals; ARRIVE guidelines recommended for reporting animal research

Laboratory animals

- Mus Musculus, C57BL/6J, from Janvier Laboratories, males, age 8-12 weeks of age
- Mus Musculus, Tg(Cd34-EGFP)MF6Gsat/Mmcd referred to as CD34-GFP mice , from in house breeding, males, age 8-12 weeks of age
- Mus Musculus, 129(Cg)-Gt(ROSA)26Sortm4(ACTB-tdTomato,-EGFP)Luo/J (referred to as mT/mG mice; (Jackson Laboratories, stock No.007676), from in house breeding, males, age 8-12 weeks of age
- Mus Musculus, CAG::KikGR33 (referred to as KikGR mice); (Jackson Laboratories, stock No. 013753), from in house breeding, males age 8-12 weeks of age

Wild animals | *The study did not involve wild animals*

Field-collected samples | *The study did not involve samples collected from the field*

Ethics oversight | *This work was submitted to and approved by the Regional Ethic Committee CEEA-122 and registered to the French Ministère de la Recherche. DAP-APAFIS-2018020110169802*

Note that full information on the approval of the study protocol must also be provided in the manuscript.

Human research participants

Policy information about studies involving human research participants

Population characteristics | All the available characteristics are presented in Table 1.

Recruitment | The recruitment criteria were: male gender, young age, active status

Ethics oversight | The protocol was approved by the Dublin City University Ethics Committee and conducted in accordance with the criteria set by the Declaration of Helsinki ; all subjects gave written informed consent.

Note that full information on the approval of the study protocol must also be provided in the manuscript.

Clinical data

Policy information about clinical studies

All manuscripts should comply with the ICMJE guidelines for publication of clinical research and a completed CONSORT checklist must be included with all submissions.

Clinical trial registration | *Provide the trial registration number from ClinicalTrials.gov or an equivalent agency.*

Study protocol | *Note where the full trial protocol can be accessed OR if not available, explain why.*

Data collection | *Describe the settings and locales of data collection, noting the time periods of recruitment and data collection.*

Outcomes | *Describe how you pre-defined primary and secondary outcome measures and how you assessed these measures.*

Dual use research of concern

Policy information about dual use research of concern

Hazards

Could the accidental, deliberate or reckless misuse of agents or technologies generated in the work, or the application of information presented in the manuscript, pose a threat to:

No	Yes	
☒	☐	Public health
☒	☐	National security
☒	☐	Crops and/or livestock
☒	☐	Ecosystems
☒	☐	Any other significant area

Experiments of concern

Does the work involve any of these experiments of concern:

No	Yes	
☒	☐	Demonstrate how to render a vaccine ineffective
☒	☐	Confer resistance to therapeutically useful antibiotics or antiviral agents
☒	☐	Enhance the virulence of a pathogen or render a nonpathogen virulent
☒	☐	Increase transmissibility of a pathogen
☒	☐	Alter the host range of a pathogen
☒	☐	Enable evasion of diagnostic/detection modalities
☒	☐	Enable the weaponization of a biological agent or toxin
☒	☐	Any other potentially harmful combination of experiments and agents

ChIP-seq

Data deposition

☐ Confirm that both raw and final processed data have been deposited in a public database such as GEO.

☐ Confirm that you have deposited or provided access to graph files (e.g. BED files) for the called peaks.

Data access links<br>*May remain private before publication.*	*For "Initial submission" or "Revised version" documents, provide reviewer access links. For your "Final submission" document, provide a link to the deposited data.*
Files in database submission	*Provide a list of all files available in the database submission.*
Genome browser session<br>(e.g. UCSC)	*Provide a link to an anonymized genome browser session for "Initial submission" and "Revised version" documents only, to enable peer review. Write "no longer applicable" for "Final submission" documents.*

Methodology

Replicates	*Describe the experimental replicates, specifying number, type and replicate agreement.*
Sequencing depth	*Describe the sequencing depth for each experiment, providing the total number of reads, uniquely mapped reads, length of reads and whether they were paired- or single-end.*
Antibodies	*Describe the antibodies used for the ChIP-seq experiments; as applicable, provide supplier name, catalog number, clone name, and lot number.*
Peak calling parameters	*Specify the command line program and parameters used for read mapping and peak calling, including the ChIP, control and index files used.*
Data quality	*Describe the methods used to ensure data quality in full detail, including how many peaks are at FDR 5% and above 5-fold enrichment.*
Software	*Describe the software used to collect and analyze the ChIP-seq data. For custom code that has been deposited into a community repository, provide accession details.*

Flow Cytometry

Plots

Confirm that:

☒ The axis labels state the marker and fluorochrome used (e.g. CD4-FITC).

☒ The axis scales are clearly visible. Include numbers along axes only for bottom left plot of group (a 'group' is an analysis of identical markers).

☒ All plots are contour plots with outliers or pseudocolor plots.

☒ A numerical value for number of cells or percentage (with statistics) is provided.

Methodology

Sample preparation: Quadriceps muscle, sub cutaneous (Sc) and perigonadic (PG) adipose tissues (AT) were harvested for cell isolation. Freshly harvested tissues were minced and stroma vascular fractions (SVF) were obtained by enzymatic digestion. PGAT and ScAT were digested with collagenase (NB4, Coger; 0,4 U/mL) and DNAse (1%, Roche) in αMEM (GIBCO) at 37°C for 45 and 60 min respectively under constant agitation. After centrifugation (300g, 10min, RT) and elimination of the floating adipocytes and the supernatant, the pellet containing the SVF was resuspended in erythrocyte lysis buffer (155 mmol/L NH_4Cl; 5,7 mmol/L K_2HPO_4; 0,1 mmol/L EDTA, pH 7.3). After filtration through 34µm sieve and centrifugation (300g, 10min, RT), cells were resuspended in autoMACS® Running Buffer (Miltenyi Biotec). Quadriceps muscles were digested with collagenase B (0,5 U/mL, Roche) and dispase II (2,4 U/mL; Roche) in Hank's Balanced Saline Solution (HBSS)+2,5 mM Ca^{2+} for 2 rounds of 30 min at 37°C under agitation separated by mechanical dissociation through G18 syringe. Reaction was stopped by adding a 2 volumes of αMEM + 10%NCS, then samples were filtered through 34µm sieve and centrifuged (300g, 10 min, RT) to eliminate supernatant and to resuspend the pellets in autoMACS® Running Buffer.

Instrument: *LSR Fortessa flow cytometer FACSDiva software (BD Biosciences).*

Software: FACSDiva software to collect data, FACSDiva and FlowLogic and FlowJo softwares to analyze flow cytometry datas.

Cell population abundance: *Our work doesn't include data on post-sort fractions.*
For flow cytometry experiments, percentage of the gated cell population is provided on the graphs.

Gating strategy: Cell suspension is first ploted on FSC/SSC where a P1 general gate is done for each tissue according to our lab expertise. Then P1 cells are ploted for FSC-H or W/ FSC-A to gate singlets ("single cells") and exclude multiple events. From there, each fluorochrome is ploted against SSC-A or FSC-A, on the isotypes samples to position negative/positive gates. We consider that the positive gate should not substantialy include events in the isotype controls. The validity of the gate position is checked on all samples (isotypes controls and marked cells). ASCs and FAPs are CD45-/CD31-/Sca-1+. Representative plots of each animal group are presented in Figures 3F, 4E, S3F.
For fluorescent ScAT-graft experiments, gating strategy for the fluorescent marker (either GFP or Tomato) was performed thanks to non-fluorescent animals. Negative/positive gates for GFP and Tomato were positionned in non-fluorescent single cells to ensure a proper negative control. GFP and Tomato positive cells amounts were measured in injured animals as well as in grafted-non-injured and non-grafted-non-injured animals as controls. Negative controls are presented in the main figures, supplemental figures or stated in the text when appropriate. Figures 3E, S3B, S3F.
For proliferation and apoptosis studies involving flow cytometry, Edu and BrdU gating has been done according to the manufacturer kit recommandations. Positive/negative gating has been done thanks to appropriate negative control animals and isotypes. Gating strategy is presented in Figure S1D for proliferation studies and in Figure 3F for apoptosis experiment.

☒ Tick this box to confirm that a figure exemplifying the gating strategy is provided in the Supplementary Information.

Magnetic resonance imaging

Experimental design

Design type: *Indicate task or resting state; event-related or block design.*

Design specifications: *Specify the number of blocks, trials or experimental units per session and/or subject, and specify the length of each trial or block (if trials are blocked) and interval between trials.*

Behavioral performance measures: *State number and/or type of variables recorded (e.g. correct button press, response time) and what statistics were used to establish that the subjects were performing the task as expected (e.g. mean, range, and/or standard deviation across subjects).*

Acquisition

Imaging type(s) | *Specify: functional, structural, diffusion, perfusion.*

Field strength | *Specify in Tesla*

Sequence & imaging parameters | *Specify the pulse sequence type (gradient echo, spin echo, etc.), imaging type (EPI, spiral, etc.), field of view, matrix size, slice thickness, orientation and TE/TR/flip angle.*

Area of acquisition | *State whether a whole brain scan was used OR define the area of acquisition, describing how the region was determined.*

Diffusion MRI ☐ Used ☐ Not used

Preprocessing

Preprocessing software | *Provide detail on software version and revision number and on specific parameters (model/functions, brain extraction, segmentation, smoothing kernel size, etc.).*

Normalization | *If data were normalized/standardized, describe the approach(es): specify linear or non-linear and define image types used for transformation OR indicate that data were not normalized and explain rationale for lack of normalization.*

Normalization template | *Describe the template used for normalization/transformation, specifying subject space or group standardized space (e.g. original Talairach, MNI305, ICBM152) OR indicate that the data were not normalized.*

Noise and artifact removal | *Describe your procedure(s) for artifact and structured noise removal, specifying motion parameters, tissue signals and physiological signals (heart rate, respiration).*

Volume censoring | *Define your software and/or method and criteria for volume censoring, and state the extent of such censoring.*

Statistical modeling & inference

Model type and settings | *Specify type (mass univariate, multivariate, RSA, predictive, etc.) and describe essential details of the model at the first and second levels (e.g. fixed, random or mixed effects; drift or auto-correlation).*

Effect(s) tested | *Define precise effect in terms of the task or stimulus conditions instead of psychological concepts and indicate whether ANOVA or factorial designs were used.*

Specify type of analysis: ☐ Whole brain ☐ ROI-based ☐ Both

Statistic type for inference (See Eklund et al. 2016) | *Specify voxel-wise or cluster-wise and report all relevant parameters for cluster-wise methods.*

Correction | *Describe the type of correction and how it is obtained for multiple comparisons (e.g. FWE, FDR, permutation or Monte Carlo).*

Models & analysis

n/a	Involved in the study
☒	☐ Functional and/or effective connectivity
☒	☐ Graph analysis
☒	☐ Multivariate modeling or predictive analysis

Functional and/or effective connectivity | *Report the measures of dependence used and the model details (e.g. Pearson correlation, partial correlation, mutual information).*

Graph analysis | *Report the dependent variable and connectivity measure, specifying weighted graph or binarized graph, subject- or group-level, and the global and/or node summaries used (e.g. clustering coefficient, efficiency, etc.).*

Multivariate modeling and predictive analysis | *Specify independent variables, features extraction and dimension reduction, model, training and evaluation metrics.*
